# A revision of the Afrotropical spider genus *Cambalida* Simon, 1909 (Araneae, Corinnidae)

**DOI:** 10.3897/zookeys.234.3417

**Published:** 2012-10-30

**Authors:** Charles Richard Haddad

**Affiliations:** 1 Department of Zoology & Entomology, University of the Free State, P.O. Box 339, Bloemfontein 9300, South Africa

**Keywords:** Castianeirinae, epigaeic, forest, new species, *nomen dubium*, savanna, taxonomy

## Abstract

The non-mimetic Afrotropical spider genus *Cambalida* Simon, 1909, placed within a subfamily of predominantly ant-mimicking spiders (Araneae: Corinnidae: Castianeirinae), is revised. Three species are transferred from *Castianeira* Keyserling, 1879 to *Cambalida*: *Cambalida deminuta* (Simon, 1909), **comb. n.**, *Cambalida fulvipes* (Simon, 1896), **comb. n.** and *Cambalida loricifera* (Simon, 1885), **comb. n.**. A fourth species, *Cambalida fagei* (Caporiacco, 1939), **comb. n.**, is transferred from *Brachyphaea* Simon, 1895 to *Cambalida*. Two species, *Castianeira depygata* Strand, 1916, **syn. n.** and *Cambalida mestrali* Lessert, 1921, **syn. n.**, are considered junior synonyms of *Cambalida fulvipes*. The males of *Cambalida deminuta* and *Cambalida loricifera* are redescribed and their unknown females are described for the first time. The female and male of *Cambalida fulvipes* and *Cambalida coriacea* Simon, 1909 are also redescribed. The type material of the type species of the genus, *Cambalida insulana* Simon, 1909 from Pagalu (Annobon) Island, is lost, and only immature specimens have been subsequently collected from a nearby island. The species is regarded as a *nomen dubium* until fresh adult material can be collected. A replacement name, *Cambalida simoni* **nom. n.** is proposed for *Cambalida fulvipes* Simon, 1909, the latter being a secondary junior homonym of *Cambalida fulvipes* (Simon, 1896). The type material of this species is also lost and it is too considered *nomen dubium*. The following new species are described: *Cambalida compressa* **sp. n.** from West Africa, *Cambalida dippenaarae* **sp. n.** from southern Africa, *Cambalida griswoldi* **sp. n.** and *Cambalida lineata* **sp. n.** from Madagascar, and *Cambalida unica* **sp. n.** from Cameroon. Notes are provided on the biology of each species and the distribution of the genus in the Afrotropical Region.

## Introduction

The spider genus *Cambalida* Simon, 1909, endemic to the Afrotropical Region, was initially described in the subfamily Micariinae of the Clubionidae by Simon (1909). Reiskind (1969) also listed this genus in the Clubionidae in his revision of North American Castianeirinae, but was uncertain of its subfamily placement. Brignoli (1983) listed the genus in the Gnaphosidae: Micariinae, while Platnick (1989) subsequently placed *Cambalida* and many of the other micariine genera in the Liocranidae. Dippenaar-Schoeman and Jocqué (1997) already listed *Cambalida* in the Corinnidae: Castianeirinae, but did not formally transfer this genus. Bosselaers and Jocqué (2000) only recently transferred *Cambalida* from the Liocranidae to the Corinnidae based on characters it shares with other members of the Castianeirinae, particularly regarding genitalic structure, a placement confirmed through subsequent phylogenetic analyses (Bosselaers and Jocqué 2002, Haddad et al. 2009).

During this study it became apparent that this small genus posed a large number of taxonomic problems that needed resolution. For example, Simon (1909) described the three species of *Cambalida* from females only, and in the same paper described four species in the genus *Castianeira* Keyserling, 1879 from males only, raising the possibility that some of these sexes could possibly be matched, which turned out not to be the case. Unfortunately, several of these types are lost, including those of the type species of *Cambalida* (*Cambalida insulana* Simon, 1909), raising problems in clarifying the taxonomic status of these species. Bosselaers and Jocqué (2000) recently redescribed *Cambalida coriacea* Simon, 1909, a species that Simon (1909) considered close to the type species but with less sclerotisation, and based on this redescription all castianeirines considered congeneric with *Cambalida coriacea* are considered in this revision to be true *Cambalida*.

The validity of *Cambalida coriacea* was recently put under threat by the discovery that *Castianeira fulvipes* Simon, 1896 may be a senior synonym. This would have resulted in a nomenclatorial change *Cambalida fulvipes* (Simon, 1896), which is a senior homonym of *Cambalida fulvipes* Simon, 1909. I proposed to the International Commission on Zoological Nomenclature that *Cambalida coriacea* have priority over its secondary senior homonym, which has been an unused name since its description (Haddad 2006). However, this proposal was rejected by Kraus (2006), and consequently *Cambalida fulvipes* (Simon, 1896) retains priority (ICZN 2007) and a new name (*Cambalida simoni* nom. n.) is proposed for the secondary junior homonym in the current study. Incidentally, the type material of this species is lost and thus *Cambalida simoni* nom. n. is considered a *nomen dubium*. Detailed examination of the genitalic morphology indicates that *Cambalida coriacea* is, in fact, a good species and that the proposed synonymy with *Cambalida fulvipes* would be incorrect, as was my earlier proposed conservation of the junior name (Haddad 2006). The present study uncovered a rich diversity of species in the Afrotropical Region, many of which have very similar male embolic structures (Figs 50–56).

**Figures 1–4.**
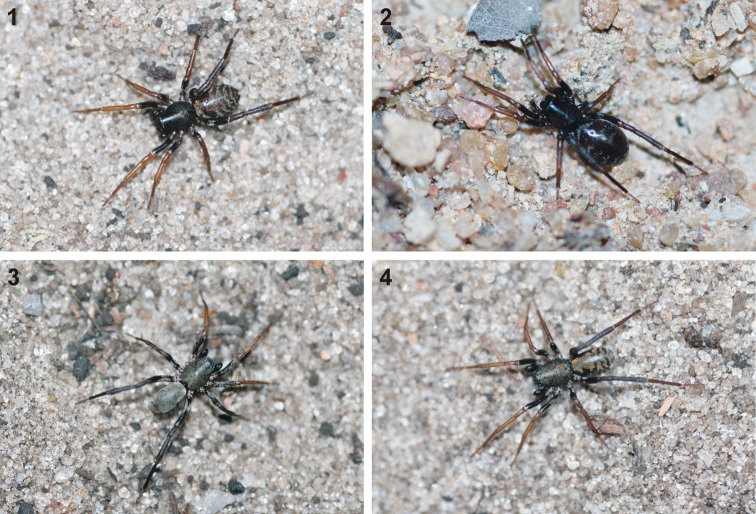
General habitus of *Cambalida dippenaarae* sp. n., indicating colour variations: **1** and **2** females and **3** male from Wildlives Game Farm, Zambia **4** male from Lesideng Research Camp, Botswana.

*Cambalida* are castianeirines with relatively unspecialised colouration (Figs 1–4), which contrasts with many genera in this subfamily that mimic ants in both colouration and behaviour. It should be noted that *Cambalida* immatures display behaviour similar to mimetic castianeirines, moving the front legs up and down to resemble antennal movements of ants. This behaviour was only rarely observed in adults. *Cambalida* are entirely ground-dwelling and are mainly associated with savanna and forest habitats on the continent, although two species occurring in southern Africa are also found in drier grassland, Nama Karoo and/or fynbos habitats.

The genus is revised here for the first time and ten species are recognised, of which five are described as new. Based on current data, *Cambalida* is considered endemic to the Afrotropical Region.

## Material and methods

Material used in this study was observed in 70% ethanol using a Nikon SMZ800 stereomicroscope for descriptions, digital photographs and measurements. The epigynes and male palps of representative specimens were dissected and cleaned in a Branson 3200 ultrasonic bath for 10 minutes in 70% ethanol, after which they were drawn. Digital photographs of the male emboli of eachspecies, as well as the dorsal habitus of *Cambalida fulvipes* and *Cambalida lineata* sp. n., were taken using a Nikon Coolpix 8400 mounted on a Nikon SMZ800 stereomicroscope. Additional photographs were taken of carapace and abdominal structures of *Cambalida fulvipes* and the holotype female of *Cambalida fagei* (Caporiacco 1939). The photographs were then stacked using Combine ZM software (http://www.hadleyweb.pwp.blueyonder.co.uk ) to increase depth of field.

Material for scanning electron microscopy was dehydrated through a graded ethanol series and then critical-point dried in an argon chamber. Specimens were then glued to aluminium stubs and sputter-coated three times with gold for 2 minutes, and subsequently studied in a JEOL WinSEM at 10kV. Digitized micrographs were taken of the morphological structures examined.

All measurements are given in millimetres (mm). Total body length measurements were determined for the smallest and largest specimens of each sex to indicate size variation, and body, eye and leg measurements are given for the specific specimens indicated. Descriptions of the eye arrangements are given for the anterior view of the anterior eye row and dorsal view of the posterior eye row.

The following abbreviations are used in the descriptions: AER – anterior eye row; AL – abdomen length; ALE – anterior lateral eye; ALS – anterior lateral spinneret(s); AME – anterior median eye; AW – abdomen width; CL – carapace length; CW – carapace width; FL – fovea length; MOQ – median ocular quadrangle; MOQAW – median ocular quadrangle anterior width; MOQL – median ocular quadrangle length; MOQPW – median ocular quadrangle posterior width; PER – posterior eye row; PERW – posterior eye row width; PLE – posterior lateral eye; PLS – posterior lateral spinneret(s); PME – posterior median eye; PMS – posterior median spinneret(s); SL – sternum length; ST – spermatheca; SW – sternum width; TL – total length.

Leg spination follows the format of Bosselaers and Jocqué (2000) and includes the following abbreviations: do – dorsal; pl – prolateral; plv – prolateral ventral; rl – retrolateral; rlv – retrolateral ventral; vt – ventral terminal.

Material used in this study is deposited in the following institutions (curators are given in parenthesis):

BMNH British Museum of Natural History, London, England (Janet Beccaloni)

CAS California Academy of Sciences, San Francisco, U.S.A. (Charles Griswold)

MNHN Museum National d’Histoire Naturelle, Paris, France (Christine Rollard)

MHNG Museum of Natural History, Geneva, Switzerland (Peter Schwendinger)

MRAC Musée Royal de l’Afrique Centrale, Tervuren, Belgium (Rudy Jocqué)

MZUF Museo di Storia Naturale, Sezione di Zoologia “La Specola”, University of Florence, Italy (Luca Bartolozzi)

NCA National Collection of Arachnida, ARC–Plant Protection Research Institute, Pretoria, South Africa (Ansie Dippenaar-Schoeman)

NMSA KwaZulu-Natal Museum, Pietermaritzburg, South Africa (Audrey Ndaba)

NMBA National Museum, Bloemfontein, South Africa (Leon Lotz)

NMZ National History Museum of Zimbabwe, Bulawayo, Zimbabwe (Moira FitzPatrick)

SAM Iziko South African Museum, Cape Town, South Africa (Margie Cochrane)

TMSA Ditsong National Museum of Natural History, Pretoria, South Africa (Robin Lyle)

ZMB Zoological Museum, Berlin, Germany (Jason Dunlop)

ZMUC Zoological Museum, University of Copenhagen, Denmark (Nikolaj Scharff)

Where depositories lacked locality co-ordinates on specimen labels, or where they were not available in the institutional databases, they were traced using the Global Gazetteer Version 2.2 (www.fallingrain.com ) and are indicated in square brackets. Distribution maps were produced using the online mapping software SimpleMappr (Shorthouse 2010).

## Taxonomy

### Family Corinnidae Karsch, 1880. Subfamily Castianeirinae Reiskind, 1969

#### Genus Cambalida

Simon, 1909

http://species-id.net/wiki/Cambalida

Cambalida insulana Simon, 1909, by original designation. *Cambalida*Simon 1909: 369; Reiskind 1969: 165; Dippenaar-Schoeman and Jocqué 1997: 128; Bosselaers and Jocqué 2000: 315. [Type species]

##### Diagnosis.

*Cambalida* is most closely related to *Castianeira* but can be recognised by the relatively broader carapace (width approximately 0.75 carapace length, usually less than 0.70 in *Castianeira*), ALE that are usually considerably larger than the AME, and the posterior eyes that are larger than those of the anterior eye row. Males can further be distinguished from all other castianeirines by the presence of two or three rows of very distinct, longer thickened setae at the distal end of the dorsal surface of the palpal cymbium (Figs 38, 39). These setae usually number between six and 10 and are sometimes accompanied by slightly shorter thickened setae to the sides of these rows. Other genera that possess thickened setae do not show such an arrangement and usually only have two or three thickened setae at the apex of the cymbium.

##### Description.

**Figures 5–13.**
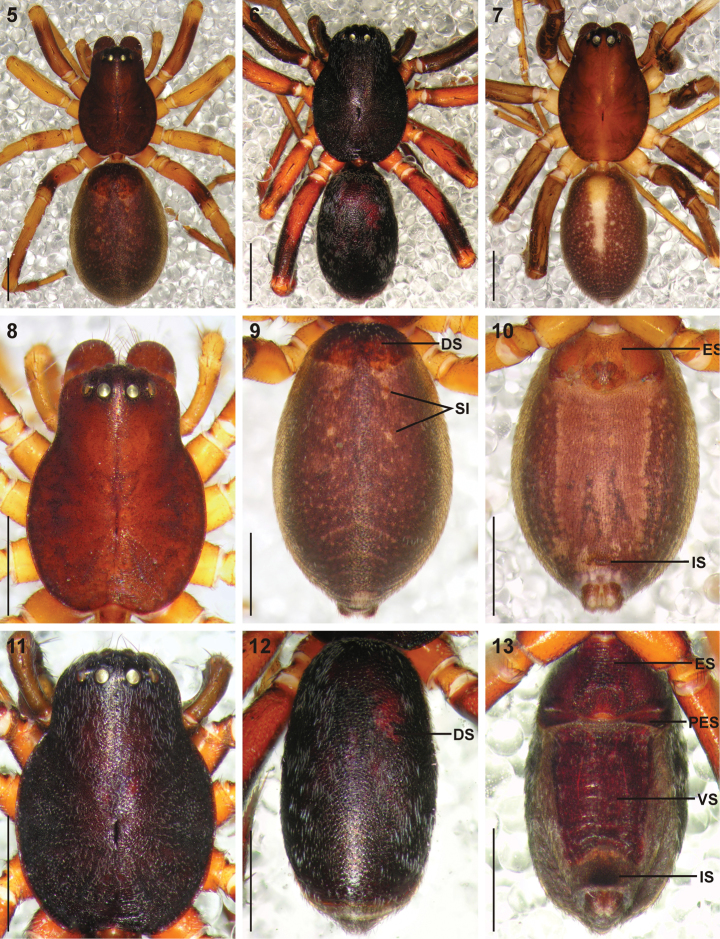
Digital microscope photographs of *Cambalida fulvipes* (Simon, 1896)from South Africa **(5, 6, 8–13)** and *Cambalida lineata* sp. n. from Madagascar **(7)**: **5, 7** female, dorsal habitus **6** male, dorsal habitus **8** female carapace, dorsal view **9** female abdomen, dorsal view **10** same, ventral view **11** male carapace, dorsal view **12** male abdomen, dorsal view **13** same, ventral view. Scale bars = 1.0mm. Abbreviations: **DS** dorsal scutum **ES  **epigastric sclerite **IS** inframamillary sclerite **PES** post-epigastric sclerite **SI** sigilla **VS** ventral sclerite.

**Figures 14–22.**
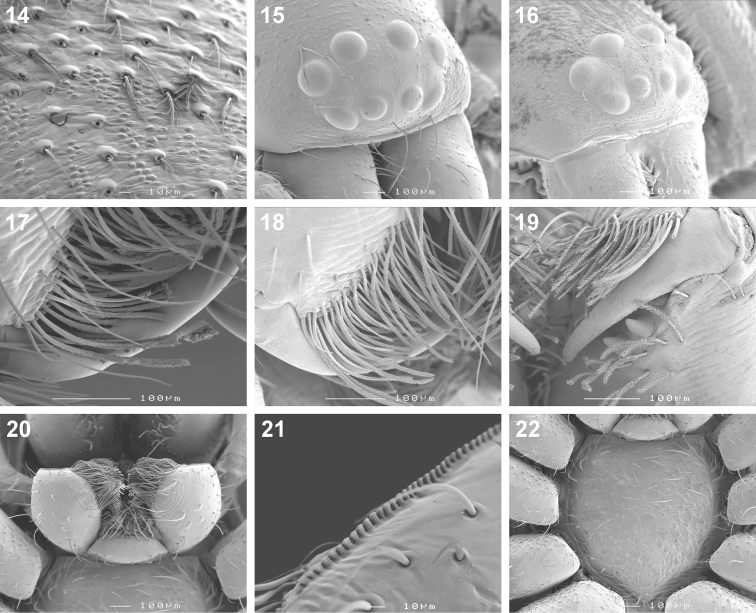
Scanning electron microscope photographs of *Cambalida dippenaarae* sp. n.female **(14, 15, 17, 20–22)** and male **(16, 18, 19)**: **14** dorsal carapace setae **15, 16** eye region and clypeus, anterolateral view **17, 18** cheliceral promarginal bent setae, anterior view **19** chelicera, ventral view **20** mouthparts, ventral view **21** serrula **22** sternum.

Small to medium sized spiders, 4.00–7.10mm in length; carapace yellow-brown to dark brown with black markings, sometimes nearly black (Figs 1–8, 11); carapace surface very finely granulate, appearing wrinkled, with scattered plumose and straight setae (Figs 8, 11, 14); several curved setae on clypeus, in eye region and posterior to PER, sometimes also along midline towards fovea (Figs 15, 16); carapace oval, broadest at coxae II, eye region narrowed; carapace slightly elevated posterior to PER, highest at one-quarter its length, depressed slightly at fovea, declining gradually behind fovea; fovea distinct, narrow, quite long; posterior margin strongly concave (Figs 8, 11). AER procurved, AME usually considerably smaller than ALE, rarely subequal in diameter; AME separated by approximately ½ their diameter, close to ALE (Figs 15, 16); PER procurved (Figs 8, 11), PME usually very slightly smaller than PLE, rarely subequal in diameter; PME closer to PLE than to each other; MOQ much wider posteriorly than anteriorly, length approximately equal to posterior width. Chilum single, triangular; anterior surface of chelicerae with scattered long and short erect straight setae; shaggy seta absent; curved setae on cheliceral promargin finely plumose in females (Fig. 17) and males (Figs 18, 19); cheliceral promargin with three teeth, retromargin with two teeth (Fig. 19); endites slightly convex laterally, with distinct serrula comprising short, slightly ventrally curved denticles (Figs 20, 21) and dense maxillar hair tuft on mesal margins (Fig. 20); labium hemispherical, nearly twice as broad as long. Pleural bars weakly sclerotised, isolated; sternum very slightly longer than broad, shield-shaped, slightly narrowed anteriorly; surface finely granulate, covered in short straight setae, with many long erect straight setae (Fig. 22); precoxal triangles and intercoxal sclerites weakly sclerotised, intercoxal sclerites only present between coxae I and II, and II and III. Leg formula 4123 in both sexes; legs finely granulate, with short spines; all segments covered in short straight black setae, with scattered black and white plumose setae (Figs 24–33), usually corresponding to markings; plumose setae sparse on tarsi; retrocoxal window on coxa I small; trochanters notched; femora usually with a single erect ventral seta proximally; patellae each with long fine distal dorsal seta (Figs 24, 25); patellar indentation narrow, slightly broadened at proximal end (Fig. 26); tibiae I and II with long do seta at ¾ tibia length, absent from tibiae III and IV; metatarsi III sometimes longer than metatarsi I and II, otherwise shorter than metatarsus I but longer than II; metatarsi scopulate distally (Fig. 29), tarsi scopulate; tibiae, metatarsi and tarsi with several dorsal and lateral trichobothria with sunken distal plate (Figs 28, 30), patellae, tibiae, metatarsi and tarsi also with several short erect setae dorsally, laterally and ventrally (Fig. 31); tarsal organ 8-shaped, slightly elevated from integument, surface finely wrinkled, opening oval and towards one side (Fig. 32); paired tarsal claws short, situated laterally, with dense claw tufts between them (Fig. 33); metatarsi III and IV without terminal preening brush or comb; palpal claw very elongate, with several ventral teeth increasing in length distally (Fig. 34). Abdomen oval, mottled grey in females, deep red with black markings in males, often with paler grey chevron markings (Fig. 9), rarely with pale median stripe (Fig. 7); three pairs of short fine straight setae on anterior margin above pedicel; dorsal scutum small and extending less than ½ abdomen length in females, covering entire dorsum in males; two pairs of distinct sclerotised dorsal sigilla present in females, absent in males (Figs 9, 12, 35); dorsum covered in short straight black setae, with scattered black and white plumose setae corresponding to chevron markings, in live specimens appearing white, yellow-brown or grey (Figs 1–4, 35); venter densely covered in plumose setae, with scattered short straight setae (Fig. 36); venter of females with moderately sclerotised epigastric region, without post-epigastric sclerites and ventral sclerite, inframamillary sclerite present, distinct, densely covered in short setae (Fig. 10); venter of males with strongly sclerotised epigastric region, post-epigastric sclerites, ventral sclerite and inframamillary sclerite, latter covered in dense short setae (Fig. 13); female with two paired rows of tiny sclerites from epigastric furrow to spinnerets, outer row weakly sclerotised and indistinct. Spinnerets (observed here in *Cambalida dippenaarae* sp. n. and by Bosselaers and Jocqué [2002] in *Cambalida coriacea*): ALS of females with two major ampullate gland spigots, many piriform gland spigots and several small nubbins (Fig. 44; Bosselaers and Jocqué 2002: fig. 9B); ALS of males with single major ampullate gland spigot, single large adjacent nubbin and many piriform gland spigots (Fig. 47; Bosselaers and Jocqué 2002: fig. 9A); PMS of females with three large cylindrical gland spigots, one small minor ampullate gland spigot and several aciniform gland spigots (Fig. 45; Bosselaers and Jocqué 2002: fig. 9D), female of *Cambalida dippenaarae* sp. n. also with a distinct tartipore; PMS of *Cambalida dippenaarae* sp. n. male with one large minor ampullate gland spigot, one tartipore and one nubbin, with several aciniform gland spigots (Fig. 48); PMS of *Cambalida coriacea* male with only a single minor ampullate gland spigot and nubbin (Bosselaers and Jocqué 2002: fig. 9C); PLS of females with two large cylindrical gland spigots and several aciniform gland spigots (Fig. 46; Bosselaers and Jocqué 2002: fig. 9F); PLS of *Cambalida dippenaarae* sp. n. male with several aciniform gland spigots and tiny nubbins (Fig. 49), of *Cambalida coriacea* male with only a single aciniform gland spigot (Bosselaers and Jocqué 2002: fig. 9E). Female epigyne weakly sclerotized, with 6-shaped or curved epigynal ridges covering or leading to lateral copulatory openings (Fig. 37); copulatory ducts directed obliquely or transversely before entering ST II along their lateral or posterior margin; ST II oval, round or subtriangular, usually connected broadly to kidney-shaped posterior ST I. Male palpal segments without apophyses; cymbium short and broad, with spines prolaterally and ventrally, covered dorsally with short straight and plumose setae; unique thickened setae arranged in two or three rows located distally on dorsal cymbium surface (Figs 38, 39); embolus situated distally, with one complete coil, breadth of base and shape of coil variable (Figs 40–43, 50–56).

**Figures 23–28. F4:**
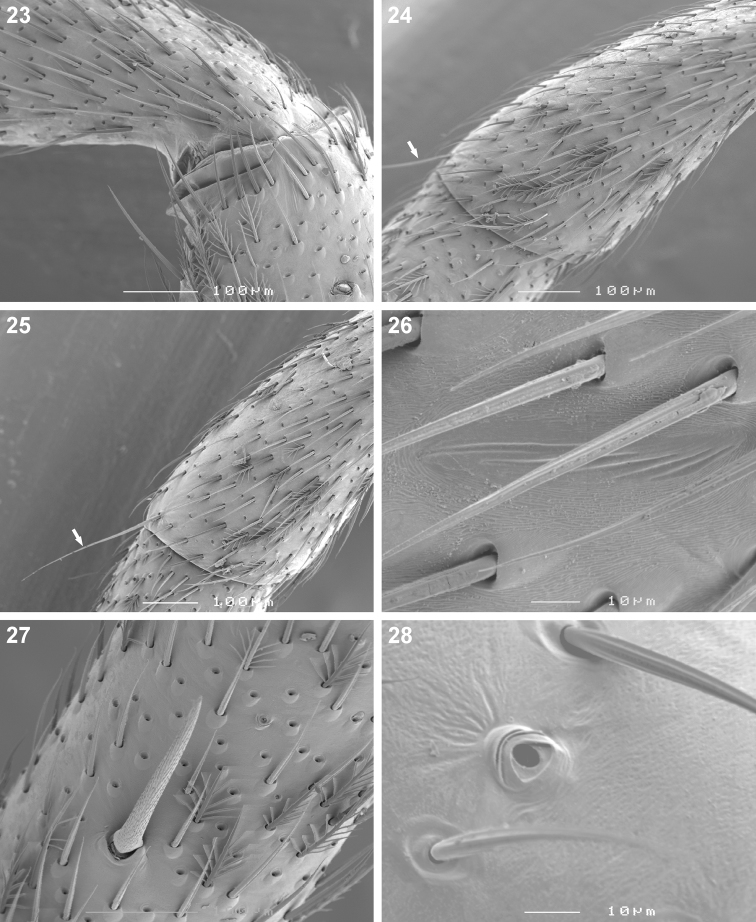
Scanning electron microscope photographs of *Cambalida dippenaarae* sp. n. female: **23**  distal end of femur IV, plumose and short straight setae **24** patella III and **25** patella IV, arrows indicating long distal setae **26** leg II, detail of lyriform organ at proximal end of patellar indentation **27** tibia IV, spine and plumose setae **28** tibia II, trichobothrium base.

**Figures 29–34. F5:**
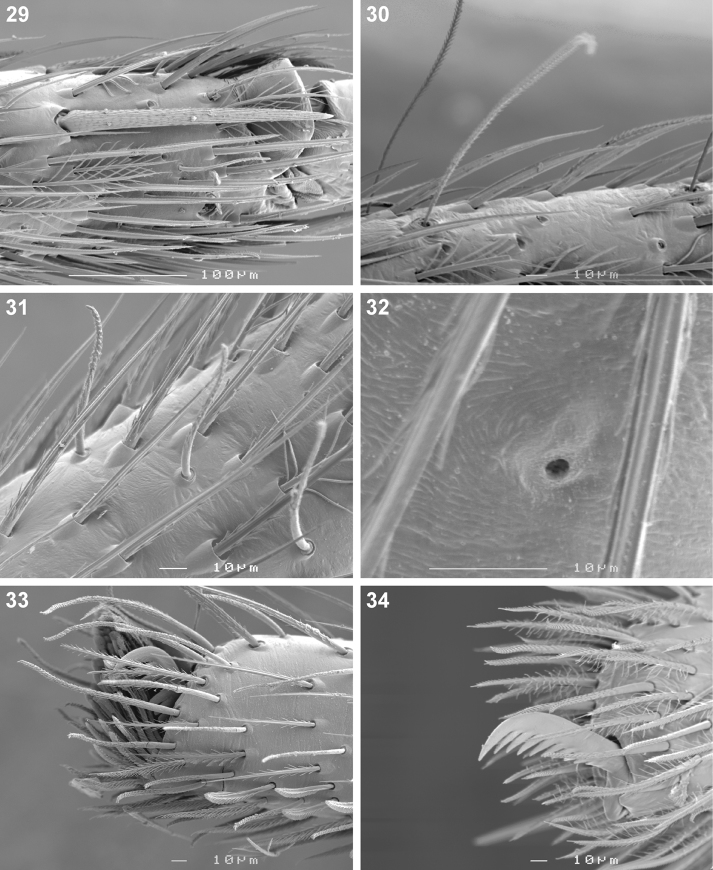
Scanning electron microscope photographs of *Cambalida dippenaarae* sp. n. female: **29** metatarsus IV, distal prolateral spine **30** tarsus IV, trichobothria **31** tarsus II, short erect setae **32** tarsus I, tarsal organ **33** same, claw tuft **34** palpal claw.

#### Cambalida compressa sp. n.

urn:lsid:zoobank.org:act:90AE7140-236F-4F0B-8E4D-FDA6A8653A58

http://species-id.net/wiki/Cambalida_compressa

Figures 40 , 50 , 57–60

##### Type material. 

**Holotype female.** NIGERIA:Niger State, Mokwa [09°17'N, 05°03'E], leg. A. Russell-Smith, 31.VIII.1974 (14 year savanna regrowth) (BMNH).

##### Paratypes.

BURKINA FASO: Bobo-Dioulasso, Matourkou, 11°05'N, 04°22'W, leg. J.O. Zongo, VIII–XII.1991 (sorghum field), 1♀ (MRAC 177035). IVORY COAST: Bouaké, F.-Foro, 07°41'N, 05°02'W, leg. G. Couturier, 19–21.VIII.1974 (piège coloré), 1♀ (MRAC 216429); Ferké poste de Comoé, Komoé River, 09°35'N, 04°20'W, leg. J. Everts, 7.III.1980, 1♀ (MRAC 173980); Mbé Research Station, West African Rice Development Association [07°52'N, 05°06'W], near Bouaké, leg. A. Russell-Smith, 17.VIII.1994 (weed control experiment), 12♂ 5♀ (BMNH); Same locality, leg. A. Russell-Smith, 1.IX.1993 (in tall *Andropogon* fallow), 2♂ 1♀ (BMNH); Touba [08°17'N, 07°41'W], leg. A. Russell-Smith, VII–X.1994 (upland rice), 14♂ 3♀ (BMNH).

##### Other material examined.

TOGO: Bassari, 09°15'N, 00°47'W, leg. P. Douben, 6.VII.1984 (pitfalls), 1♂ (MRAC 173987).

##### Diagnosis.

The females are easily recognised by the very small spermathecae, large 6-shaped epigynal ridges and copulatory ducts that are initially directed medially (Figs 57, 58). Males have an embolus that is distinctly compressed on its longitudinal axis (Figs 40, 50, 59).

##### Etymology.

From the Latin for compact, compressed, referring to the structure of the male embolus.

##### Remark.

The majority of the specimens examined have a much lighter colouration than their congeners, as described below, i.e. a yellow carapace and legs and yellow abdomen with black mottling. The remaining specimens had a brown body and legs with similar markings to the majority of congeners. Since the genitalic morphology is very stable in all of the specimens examined it is clear that this species is a case of colour polymorphism.

##### Female

**(holotype, Mokwa, BMNH).** Measurements: CL 2.69, CW 1.84, AL 3.60, AW 2.10, TL 6.40 (5.80–7.00), FL 0.22, SL 1.19, SW 1.11, AME–AME 0.06, AME–ALE 0.02, ALE–ALE 0.38, PME–PME 0.11, PME–PLE 0.06, PLE–PLE 0.52, PERW 0.79, MOQAW 0.37, MOQPW 0.48, MOQL 0.49.

Length of leg segments (sequence from femur to tarsus, and total): I 2.10 + 0.85 + 1.78 + 1.66 + 1.20 = 7.59; II 1.95 + 0.78 + 1.45 + 1.54 + 1.07 = 6.79; III 1.80 + 0.75 + 1.38 + 1.68 + 0.95 = 6.56; IV 2.63 + 0.90 + 2.30 + 1.73 + 0.77 = 8.33.

Carapace dark orange-brown with black mottling, clypeus mottled black medially and yellow-brown laterally, eye region black; black striae radiating from fovea towards palps and leg coxae; surface finely granulate, sparsely covered in white plumose setae. All eyes with black rings; AER procurved, ALE larger than AME; AME separated by distance slightly less than ½ their diameter, AME separated from ALE by distance slightly less than ^1^∕_5_ AME diameter; clypeus height slightly larger than AME diameter; PER procurved, PLE slightly larger than PME; PME separated by distance equal to ^3^∕_5_ their diameter, PME separated from PLE by distance slightly less than ⅓ PME diameter; CW:PERW = 2.33:1. Chelicerae orange-brown with faint black mottling on anterior surface, orange proximally and along prolateral distal margin; three teeth on promargin, median tooth largest, proximal and distal teeth subequal, distal tooth situated closest to median tooth; two slightly separated subequal teeth on retromargin, closer to fang base than promarginal teeth; endites yellow-brown with faint black mottling, cream prolaterally; labium yellow-brown, cream distally; sternum bright yellow with faint black mottling. Legs finely granulate; legs I–III uniform yellow with black mottling laterally, except on tarsi; femora IV yellow with black mottling; patellae IV yellow with black mottling faint proximally, dark in distal half; tibiae IV yellow-brown with black mottling, yellow at distal end; metatarsi IV yellow-brown with black mottling, yellow proximally and distally; tarsi IV yellow. Leg spination: femora: I pl 1 do 3, II pl 1 do 3, III pl 2 do 3 rl 2, IV pl 2 do 3 rl 1; patellae with do 1 long distal seta; tibiae: I plv 2 rlv 2, II plv 1 rlv 3, III pl 2 do 1 rl 2 plv 2 rlv 2 vt 2, IV pl 2 do 1 rl 2 plv 2 rlv 2 vt 2; metatarsi: I plv 2 rlv 2, II plv 2 rlv 2, III pl 3 rl 3 plv 2 rlv 2 vt 3, IV pl 3 rl 3 plv 2 rlv 2 vt 3. Palpal spination:  femora pl 1 do 2, patellae pl 1 do 1, tibiae pl 1 do 2 plv 1, tarsi pl 1 plv 3 rlv 1. Abdomen lilac-grey, with indistinct white chevrons of plumose setae and two small white patches above spinnerets; dorsal scutum pale brown, extending only ^1^∕_10_ abdomen length; venter pale lilac-grey with cream mottling, epigastric sclerite and inframamillary sclerite yellow-brown. Epigyne with large lateral 6-shaped epigynal ridges with prolateral copulatory openings (Fig. 57); copulatory ducts directed medially, curving anteriorly, entering ST II posterolaterally; ST II small and oval, joined broadly to narrow kidney-shaped posterior ST I (Fig. 58).

**Figures 35–43.**
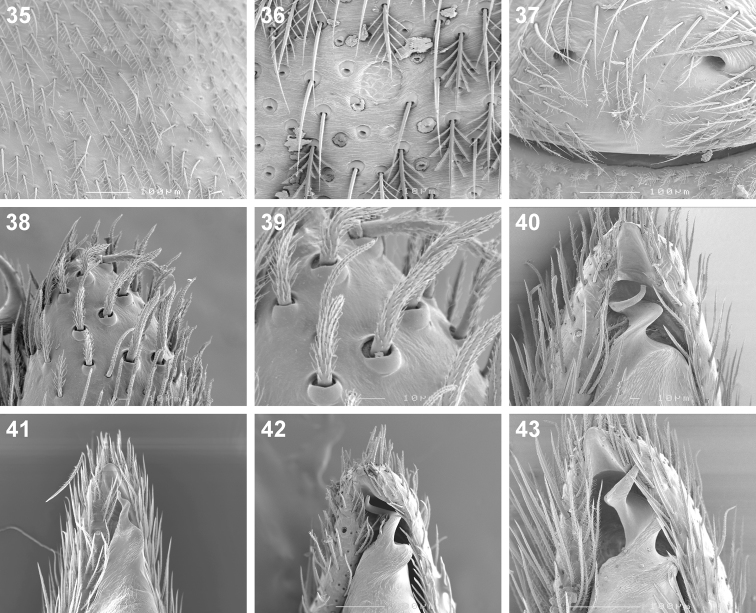
Scanning electron microscope photographs of *Cambalida dippenaarae* sp. n. **(35–39, 42)**, *Cambalida compressa* sp. n. **(40)**, *Cambalida deminuta* (Simon, 1909) **(41)** and *Cambalida loricifera* (Simon, 1885) **(43)**: **35** female, dorsal abdominal surface **36** dorsal abdominal sigillum and detail of plumose setae **37** female epigyne **38** thickened setae at dorsal distal end of male palpal cymbium **39** detail of modified setae **40–43** male emboli.

##### Male

**(paratype, Mbé, BMNH).** Measurements: CL 2.50, CW 1.75, AL 2.50, AW 1.30, TL 5.00 (4.15–5.75), FL 0.17, SL 1.11, SW 1.02, AME–AME 0.05, AME–ALE 0.01, ALE–ALE 0.33, PME–PME 0.09, PME–PLE 0.04, PLE–PLE 0.48, PERW 0.73, MOQAW 0.35, MOQPW 0.43, MOQL 0.46.

Length of leg segments (sequence from femur to tarsus, and total): I 2.18 + 0.71 + 1.83 + 1.82 + 1.35 = 7.89; II 1.92 + 0.74 + 1.55 + 1.60 + 1.13 = 6.94; III 1.82 + 0.68 + 1.43 + 1.77 + 0.97 = 6.67; IV 2.73 + 0.83 + 2.41 + 2.92 + 1.33 = 10.22.

Carapace bright yellow with faint black mottling, yellow-brown in cephalic region, clypeus mottled black medially, yellow-brown laterally, eye region black; faint black striae radiating from fovea towards palps and leg coxae; surface finely granulate, densely covered in white plumose setae. All eyes with black rings; AER procurved, ALE very slightly larger than AME; AME separated by distance slightly less than ⅓ their diameter, AME separated from ALE by less than ^1^∕_10_ AME diameter; clypeus height slightly larger than AME diameter; PER procurved, PME very slightly smaller than PLE; PME separated by distance ½ their diameter, PME separated from PLE by distance slightly less than ¼ PME diameter; CW:PERW = 2.40:1. Chelicerae yellow-brown with black mottling on anterior surface, yellow along prolateral distal margin; three teeth on promargin, median tooth largest, proximal tooth slightly smaller than distal tooth, distal tooth situated closest to median tooth; two slightly separated teeth on retromargin, distal tooth slightly smaller than proximal tooth, closer to fang base than promarginal teeth; endites pale yellow-brown with faint black mottling, cream prolaterally; labium pale orange-brown with faint black mottling, cream distally; sternum yellow with black mottling. Legs finely granulate; legs I–III pale yellow with faint black mottling dorsally and laterally; femora IV pale yellow with faint black mottling; patellae IV yellow with black mottling laterally and ventrally, faint dorsally; tibiae IV yellow with black mottling laterally and ventrally, faint dorsally, distal end pale yellow; metatarsi IV pale yellow with black mottling, faint distally, absent proximally; tarsi IV creamy-yellow. Leg spination: femora: I pl 1 do 3, II pl 1 do 3, III pl 2 do 3 rl 2, IV pl 2 do 3 rl 2; patellae with do 1 long distal seta; tibiae: I plv 3 rlv 3, II plv 1 rlv 3, III pl 2 do 1 rl 2 plv 2 rlv 2 vt 2, IV pl 2 do 1 rl 2 plv 2 rlv 2 vt 2; metatarsi: I plv 2 rlv 2, II plv 2 rlv 2, III pl 3 rl 3 plv 2 rlv 2 vt 3, IV pl 3 rl 3 plv 2 rlv 2 vt 3. Palpal spination: femora pl 1 do 2, patellae pl 1 spine do 2 setae, one proximally and one distally, tibiae pl 1 plv 1, tarsi pl 2 plv 2. Abdomen with pale orange-brown dorsal scutum with faint black mottling, nearly covering entire dorsum, with small white spot of dense plumose setae just above spinnerets; sides of abdomen mottled dark grey; venter creamy grey, epigastric sclerite and post-epigastric sclerites yellow, ventral sclerite creamy-yellow, inframamillary sclerite yellow-brown. Palps yellow with faint black mottling; embolus short and compressed on its longitudinal axis, with one and a quarter coils, tip directed retrolaterally distally (Figs 40, 50, 59, 60).

##### Distribution.

Widespread in West Africa but only known from a few scattered localities (Fig. 65).

##### Biology.

Several records come from agroecosystems (fallow, sorghum and rice); the rest are from riparian forest and savanna habitats.

#### Cambalida coriacea

Simon, 1909

http://species-id.net/wiki/Cambalida_coriacea

Figures 51 , 61–64

Cambalida coriacea Simon 1909: 370; Bosselaers and Jocqué 2000: 315, figs 3D, 4A–H; Bosselaers and Jocqué 2002: 244, figs 9A–F.

##### Type material.

**Holotype female.** SIERRA LEONE: Free Town [08°29'N, 13°14'W], MNHN 24399 (examined).

##### Other material examined.

CAMEROON: Chabal Mbabo, SW Slope, 07°25'N, 12°49'E, 1200m a.s.l., leg. Bosmans & Van Stalle, 8.IV.1983 (gallery forest, litter), 1♀ (MRAC 162248); Same locality, 1250m a.s.l., leg. Bosmans & Van Stalle, 7–13.IV.1983 (gallery forest, pitfalls), 3♂ (MRAC 162198); Same locality, 1400m a.s.l., leg. Bosmans & Van Stalle, 11.IV.1983 (gallery forest, litter), 1imm. 2♂ (MRAC 162202); Same locality, 1600m a.s.l., leg. Bosmans & Van Stalle, 10.IV.1983 (transition gallery forest to grassland, sweep-net), 1♂ (MRAC 162204); Same locality, 1600m a.s.l., leg. Bosmans & Van Stalle, 7–13.IV.1983 (gallery forest, pitfalls), 1♂ (MRAC 162213); Faro Game Reserve, 08°24'N, 12°49'E, leg., R. Jocqué, K. Loosveldt, L. Baert & M. Alderweireldt, 27.IV.2007 (mature gallery forest, sieving), 1♂ (MRAC 221324); Matute, Tiko Plantation [04°04'N, 09°21'E], leg. B. Malkin, 24.IV–6.V.1949, 1♀ (CAS, CASENT 9033096); Mbam mountain area, near Koutoupi, W slope, 05°54'N, 10°44'E, 1500m a.s.l., leg. Bosmans & Van Stalle, 30.III.1983 (gallery forest), 1♀ (MRAC 162196); Same locality, 1950m a.s.l., leg. Bosmans & Van Stalle, 30.III–3.IV.1983 (grassland), 1imm. 1♀ (MRAC 162197); Same locality, 1580m a.s.l., leg. Bosmans & Van Stalle, 30.III.1983 (transition gallery forest to grassland), 1♀ (MRAC 162242); Same locality, 1580m a.s.l., leg. Bosmans & Van Stalle, 30.III.1983 (transition gallery forest to grassland, pitfalls), 1♂ (MRAC 162201); Same locality, 1100m a.s.l., leg. Bosmans & Van Stalle, 31.III.1983 (forest, litter), 1♂ (MRAC 162209). D.R. CONGO: *North Kivu*: Parc National Albert, sector Tshiaberimu, Riv. Talya Nord afl. Semliki, 01°13'N, 30°32'E, 2340m a.s.l., leg. P. Vanschuytbroeck & H. Synave, 26.III.1954 (Berlese), 1♂ (MRAC 216076), 1♂ (MRAC 216101). *Tshopo*: Kisangani, forêt de Masako, 00°35'N, 25°11'E, leg. J.-L. Juakaly, 17.XII.2002 (young fallow, pitfall), 2♂ (MRAC 214425); Same locality, leg. J.-L. Juakaly, 11.III.2003 (secondary forest of 40 years old, pitfall), 1♂ (MRAC 214426); Same locality, leg. J.-L. Juakaly, 2.VII.2002 (old fallow, pitfall), 1imm. 2♂ (MRAC 214427); Same locality, leg. J.-L. Juakaly, 24.IX.2002 (young secondary forest, pitfall), 1♂ (MRAC 214580); Kisangani, University campus, 00°31'N, 25°11'E, leg. R. Jocqué, 13.XII.2007 (garden, by hand), 1♂ (MRAC 222506). GABON: Woleu-Ntem, Assok-Ngum, 01°45'N, 11°39'E, leg. A. Pauly, 24.II.1986 (coupe forestière, piège eau), 2♂ (MRAC 172865). GHANA: Kakum Forest, 05°20'N, 01°23'W, 159m a.s.l., leg. R. Jocqué, D. de Bakker & L. Baert, 16.XI.2005 (sieving litter, secondary forest), 1♂ (MRAC 217222). GUINÉE: Forêt classée de Ziama, 08°24'N, 09°17'W, leg. D. Flomo, 21.I.1999 (rain forest, pitfalls), 1♂ (MRAC 218217); Same data, 17.II.2000, 1♂ (MRAC 218219); Same data, 26.IV.1999, 1♀ (MRAC 217955); Same data, 30.VI.1999, 1♂ (MRAC 218220); Same data, 4.II.2000, 1♂ (MRAC 218221); Same data, 15.II.1999, 2♂ (MRAC 218222). IVORY COAST: Abengourou, Forêt classée de Bossematié, 06°37'N, 03°27'W, leg. M. Mühlenberg, 12.III.1993 (rain forest), 1♂ (MRAC 177079); Appouesso, Forêt classée de la Bossematié, 06°35'N, 03°28'W, leg. R. Jocqué & N. Séabé, 30.XI.1994 (rain forest), 1♂ (MRAC 202965); Same locality, leg. R. Jocqué, 1.XII.1994 (modified Malaise trap), 1♂ (MRAC 200938); Same locality, leg. R. Jocqué & Tanoh, 12.III.1995 (forest, pitfall), 1♀ (MRAC 204297); Same data, 26.III.1995, 1♀ (MRAC 204288); Same data, 9.IV.1995, 1♂ (MRAC 204284); Same data, 23.IV.1995, 1♂ (MRAC 204302); Same data, 7.V.1995, 1♂ (MRAC 204283); Same data, 20.V.1995, 1♀ (MRAC 204299), 1♀ (MRAC 204301), 1♂ (MRAC 204303), 1♂ 1♀ (MRAC 204304); Same data, 4.VI.1995, 1♂ (MRAC 204282), 1♀ (MRAC 204300), 1♂ (MRAC 204291); Same data, 18.VI.1995, 1♂ (MRAC 204295), 1♂ (MRAC 204298); Same data, 2.VII.1995, 2♂ 1♀ (MRAC 204285); Same data, 5.XI.1995, 1♂ (MRAC 204296), 1♂ (MRAC 204294); Same data, 19.II.1997, 1♀ (MRAC 205389); Bouaflé, 06°59'N, 05°45'W, leg. J. Everts, 12.I.1981 (pitfalls), 1♂ 5♀ (MRAC 174000); Bouaké, F.-Foro, 07°41'N, 05°02'W, leg. G. Couturier, 5–7.VIII.1974 (piège coloré), 1♀ (MRAC 216372); Guiglo [06°32'N, 07°29'W], leg. Verheyen & Thys van den Audenaerde, 5.VIII.1966, 1♀ (MRAC 131446); Mankono, Ranch de la Marahoué, 08°27'N, 06°52'W, leg. J. Everts, I.1980 (riverine forest), 1♂ (MRAC 172274); Same data, II.1980, 1♂ (MRAC 172249); Same data, III.1980, 8♂ 1♀ (MRAC 172252); Same data, III.1980, 1♂ (MRAC 172261); Touba [08°16'N, 07°41'W], leg. A. Russell-Smith, VII–X.1994, 3♀ (PCRS). LIBERIA: Bong Range Forest, 06°49'N, 10°17'W, leg. D. Flomo, 30.V.2005 (pitfalls in rain forest), 1♀ (MRAC 217132). NIGERIA: Lagos State, Iseri [Isheri, 06°38'N, 03°23'E], leg. B. Malkin, 9–10.IV.1949, 1♀ (CAS, CASENT 9033092). UGANDA: Rubaga, 00°18'N, 32°33'E, leg. D. Penney, VI.1994 (pitfall traps in compound), 1♂ (MRAC 219541).

##### Diagnosis.

Females can be recognised by the relatively long entrance ducts that are initially directed anterolaterally before looping posteriorly, medially and then anteriorly before entering triangular ST II (Figs 61, 62). Males have an embolus with a triangular basal section, and distal section of the coil narrow and tapering to a sharp point (Figs 51, 63, 64).

##### Female

**(Matute, CASENT 9033096).** Measurements: CL 2.04, CW 1.53, AL 2.50, AW 1.80, TL 4.60 (4.50–5.68), FL 0.20, SL 0.94, SW 0.91, AME–AME 0.06, AME–ALE 0.02, ALE–ALE 0.29, PME–PME 0.10, PME–PLE 0.04, PLE–PLE 0.44, PERW 0.65, MOQAW 0.28, MOQPW 0.39, MOQL 0.38.

Length of leg segments (sequence from femur to tarsus, and total): I 1.53 + 0.62 + 1.24 + 1.22 + 0.80 = 5.41; II 1.43 + 0.60 + 1.06 + 1.11 + 0.73 = 4.93; III 1.25 + 0.57 + 0.95 + 1.20 + 0.65 = 4.62; IV 1.83 + 0.67 + 1.60 + 1.90 + 0.77 = 6.77.

Carapace deep orange-brown, clypeus yellow, eye region darker; mottled black striae radiating from fovea towards palps and leg coxae; surface finely granulate, sparsely covered in white plumose setae. All eyes with black rings; AER procurved, ALE much larger than AME; AME separated by distance slightly more than ½ their diameter, AME separated from ALE by distance slightly more than ⅛ AME diameter; clypeus height slightly larger than 1¼ AME diameter; PER procurved, PME slightly larger than PLE; PME separated by distance equal to ^3^∕_5_ their diameter, PME separated from PLE by distance equal to ¼ PME diameter; CW:PERW = 2.35:1. Chelicerae pale orange-brown, with black mottling on anterior surface; three teeth on promargin, median tooth largest, distal tooth smallest, situated closest to median tooth; two teeth on retromargin, separated by their basal width, distal tooth slightly smaller than proximal tooth, closer to fang base than promarginal teeth; endites yellow with faint black mottling, cream prolaterally; labium yellow-brown, cream distally; sternum pale orange-brown with black mottling. Legs finely granulate; femora I–IV brown, yellow proximally and distally, with paired yellow stripes dorsally in distal half; patellae I–IV yellow, with faint mottled brown prolateral spot, retrolateral end fringed with brown, marking extending around patellar indentation; tibiae, metatarsi and tarsi I and II yellow, with sparse brown mottling; tibiae III yellow with ventral brown band in distal third; tibiae IV yellow with broad brown median band and paired yellow stripes dorsally; metatarsi and tarsi III and IV yellow with faint brown mottling. Leg spination: femora: I pl 1 do 3, II pl 1 do 3, III pl 2 do 3 rl 1, IV pl 1 do 3 rl 1; patellae with do 1 long distal seta; tibiae: I plv 1 rlv 1, II rlv 1, III pl 2 rl 2 plv 1 vt 2, IV pl 2 rl 2 plv 2 vt 2; metatarsi: I plv 2 rlv 2, II plv 2 rlv 2, III pl 2 rl 2 plv 2 rlv 2 vt 3, IV pl 3 rl 3 plv 2 rlv 2 vt 3. Palp with femora and patellae yellow, tibiae and tarsi yellow-orange, all with black mottling. Palpal spination: femora do 2, patellae pl 1 do 2, tibiae pl 1 do 1 plv 1, tarsi pl 1 plv 3 rlv 1. Abdomen mottled dark grey, with darker median stripe and orange dorsal scutum extending ¼ abdomen length; small white spot of dense plumose setae just above spinnerets; venter creamy-grey, darker towards spinnerets, epigastric sclerite and inframamillary sclerite orange-brown. Epigyne with lateral copulatory openings situated narrow curved epigynal ridges (Fig. 61); copulatory ducts initially directed anterolaterally, looping posteriorly and then transversely medially, bending sharply before entering subtriangular ST II posterolaterally; ST II joined broadly to kidney-shaped posterior ST I (Fig. 62).

##### Male

**(Ibadan, BMNH).** Measurements: CL 2.50, CW 1.84, AL 2.80, AW 1.65, TL 5.15 (4.90–5.40), FL 0.21, SL 1.10, SW 1.03, AME–AME 0.06, AME–ALE 0.02, ALE–ALE 0.32, PME–PME 0.11, PME–PLE 0.04, PLE–PLE 0.51, PERW 0.75, MOQAW 0.33, MOQPW 0.46, MOQL 0.45.

Length of leg segments (sequence from femur to tarsus, and total): I 2.00 + 0.75 + 1.78 + 1.85 + 1.22 = 7.60; II 1.78 + 0.73 + 1.40 + 1.57 + 1.00 = 6.48; III 1.67 + 0.70 + 1.25 + 1.65 + 0.82 = 6.09; IV 2.38 + 0.80 + 2.12 + 2.70 + 1.05 = 9.05.

Carapace bright orange-brown, clypeus bright yellow with black mottling medially, eye region with dense black mottling; black striae radiating from fovea towards palps and leg coxae; surface finely granulate, sparsely covered in white plumose setae. All eyes with black rings; AER procurved, ALE much larger than AME; AME separated by distance approximately ^2^∕_5_ their diameter, AME separated from ALE by ⅛ AME diameter; clypeus height equal to 1^2^∕_5_ AME diameter; PER procurved, PME and PLE equal in diameter; PME separated by distance equal to ^3^∕_5_ their diameter, PME separated from PLE by distance slightly more than ^1^∕_5_ PME diameter; CW:PERW = 2.45:1. Chelicerae bright yellow-orange with black mottling on anterior surface, except distally; three teeth on promargin, median tooth largest, distal tooth slightly smaller than proximal tooth, distal tooth situated closest to median tooth; two slightly separated subequal teeth on retromargin, closer to fang base than promarginal teeth; endites yellow with black mottling, cream prolaterally; labium yellow-orange proximally, cream distally; sternum bright orange with black mottling. Legs finely granulate; legs I–III creamy-yellow, IV bright yellow, femora slightly darker, all with faint black mottling laterally. Leg spination: femora: I pl 1 do 3, II pl 1 do 3, III pl 2 do 3 rl 1, IV pl 2 do 3 rl 1; patellae with do 1 long distal seta; tibiae: I plv 1 rlv 1, II rlv 2, III pl 2 do 1 rl 2 plv 2 rlv 2 vt 2, IV pl 2 do 1 rl 2 plv 2 rlv 1 vt 2; metatarsi: I plv 2 rlv 2, II plv 2 rlv 2, III pl 3 rl 3 plv 2 rlv 2 vt 3, IV pl 3 rl 3 plv 2 rlv 2 vt 3. Palpal spination: femora pl 1 do 2, patellae pl 1 spine do 2 short setae, tibiae pl 1 plv 1, tarsi pl 2 plv 2. Abdomen with dark orange-brown dorsal scutum with dense black mottling, extending ⅞ abdomen length, with small white spot of dense plumose setae just above spinnerets; posterior end of dorsum and sides of abdomen pale grey; venter pale grey, epigastric sclerite, post-epigastric sclerites and ventral sclerite orange-brown, inframamillary sclerite pale yellow-brown. Palps creamy-yellow, tarsi yellow, with faint black mottling; embolus forming a narrow coil, with a triangular basal section and distal section of the coil tapering to a sharp point (Figs 51, 63, 64).

##### Distribution.

Widespread in central and western Africa (Fig. 65).

##### Biology.

This species has been collected from a variety of tropical forest types, mainly by pitfall trapping, litter sifting and by hand.

#### Cambalida deminuta

(Simon, 1909)

http://species-id.net/wiki/Cambalida_deminuta

Figs 41 , 52 , 66–69

Castianeira deminuta Simon, 1909: 367 comb. n.

##### Type material.

**Male lectotype and male paralectotype, here designated**, together with 1 male *Cambalida fulvipes* (Simon, 1896). D.R. CONGO: Fernand Vaz, deposited in MNHN 4109 (examined).

##### Other material examined.

ANGOLA: Chimporo [17°20'S, 17°17'E], 1♂ (MNHG); Vila Luso [Luene, 11°46'S, 19°55'E], leg. B. Malkin, 24–25.XI.1949, 1♂ (CAS, CASENT 9033128). CAMEROON: Muyuka [04°17'N, 09°24'E], leg. B. Malkin, 24–29.VI.1949, 1♀, together with 1♂ *Cambalida fulvipes* (Simon, 1896) (CAS, CASENT 9033116). CENTRAL AFRICAN REPUBLIC: Bambari, 04°15'N, 21°54'E, leg. G. Pierrard, II.1969, 1♂ (MRAC 136621). D.R. CONGO: *Bas-Congo*: Mayombe, Luki Forest Reserve, 05°37'S, 13°05'E, leg. W. Hubau, 18–19.IX.2007 (caught by hand, along trail near guest house), 1♀ (MRAC 222143). *Kivu*: Butembo, 00°07'N, 29°17'E, leg. M. Lejeune, VI.1971, 1♀ (MRAC 140877); Butembo, Vallée Musosa [00°17'N, 29°45'E], 1745m a.s.l., leg. M. Lejeune, IV.1968, 1♀ (MRAC 134048); Lubero, grotte Ribue Lya Mikako [00°09'S, 29°13'E], 1500m a.s.l., leg. J. Celis & M. Lejeune, 27.XII.1966, 1♀ (MRAC 131337). *Tshopo*: Kisangani, Forêt de Masako, 00°35'N, 25°11'E, leg. J. Juakaly, 12.VII.2001 (old Hevea plantation), 1♀ (MRAC 212037); Same data, 18.VII.2001, 1♀ (MRAC 211823); Same data, 20.VII.2001, 1♀ (MRAC 211833); Same locality, leg. J.-L. Juakaly, 6.I.2003 (night catch, young fallow), 1♀ (MRAC 214691), 1♀ (MRAC 214700); Same locality, leg. J.-L. Juakaly, 2.VII.2002 (pitfalls, young fallow), 1♂ (MRAC 214346), 1♂ (MRAC 214421), 2♂ (MRAC 214423); Same data, 17.XII.2002, 1♀ (MRAC 214422), 2♂ (MRAC 214424); Same data, 24.IX.2002, 2♂ (MRAC 214577), 2♂ (MRAC 214578), 1♀ (MRAC 214579); Same locality, leg. J.-L. Juakaly, 11.III.2002 (pitfalls, young secondary forest), 1♂ (MRAC 214428), 1♀ (MRAC 214429); Same data, 5.VII.2001, 1♀ (MRAC 212083); Same locality, leg. J. Juakaly, 4.VII.2001 (young fallow), 1♀ (MRAC 211916); Same data, 11.VII.2001, 1♀ (MRAC 212069), 1♂ (MRAC 212094); Same data, 12.VII.2001, 1♂ 2♀ (MRAC 212053); Same data, 18.VII.2001, 2♀ (MRAC 211850); Same data, 20.VII.2001, 2♀ (MRAC 211826), 1♂ (MRAC 211827); Same data, 25.VII.2001, 1♂ 3♀ (MRAC 211803), 1♀ (MRAC 211815); Same data, 26.VII.2001, 1imm. 4♂ 1♀ (MRAC 211788); Same data, 27.VII.2001, 1♀ (MRAC 211841). GABON: Province Estuaire, Pointe Ngombe, Ekwata, 16km 240° WSW Libreville, 5m a.s.l., 00°19'27"N, 09°18'43"E, leg. B.L. Fisher, 27.III.2000 (littoral rainforest, sifted litter), 1♀ (CAS BLF #2294). GUINÉE: F.C. de Ziama, 08°24'N, 09°17'W, leg. D. Flomo, 18.III.2000 (pitfalls, rain forest), 1♀ (MRAC 217954); Same data, 13.IV.1999, 1♀ (MRAC 217953); Same data, 26.IV.1999, 1♀ (MRAC 217956); Same data, 4.VI.1999, 1♀ (MRAC 217957). IVORY COAST: Appouesso, F.C. Bossematié, 06°35'N, 03°28'W, leg. R. Jocqué, 21.XI.1994 (rain forest, grappe 10, near fallen tree), 1♀ (MRAC 201096); Same locality, leg. R. Jocqué & Tanoh, 4.VI.1995 (pitfalls, forest), 1♂ (MRAC 204289); Same data, 18.VI.1995, 1♀ (MRAC 204286); Same data, 2.VII.1995, 1♀ (MRAC 204287); Same data, 30.VII.1995, 1♀ (MRAC 204293); Same locality, Route no. 1, 06°35'N, 03°28'W, leg. R. Jocqué & L. Baert, 19.II.1997 (rain forest, by night, layon 19), 1♀ (MRAC 205436); Bouaké, F.-Foro, 07°41'N, 05°02'W, leg. G. Couturier, 12–14.VIII.1974 (piège coloré), 1♂ (MRAC 216487); Same data, 19–21.VIII.1974, 1♂ (MRAC 216409); Mankono, Ranch de la Marahoué, 08°27'N, 06°52'W, leg. J. Everts, III.1980 (riverine forest), 1♂ (MRAC 172259). LIBERIA: Bong Range Forest, 06°49'N, 10°17'W, leg. D. Flomo, 8.IV.2005 (pitfalls in rain forest), 1♀ (MRAC 216650). RWANDA: Parc National Akagera, Lake Ihema, pêcherie, 01°55'S, 30°45'E, leg. Jocqué, Nsengimana & Michiels, 14.XI–8.XII.1985, 1♀ (MRAC 164699); Same locality, 6km S of Lake Ihema, leg. Jocqué, Nsengimana & Michiels, 30.XI–7.XII.1985 (Berlèse), 1♀ (MRAC 164807). TOGO: Bassari, 09°15'N, 00°47'W, leg. P. Douben, V–VII.1984 (pitfalls), 1♂ (MRAC 173983). UGANDA: Kanyawara, 00°34'N, 30°21'E, 1600m a.s.l., V. & B. Roth, 30.X.1992, 1♂, together with 3 imm. *Castianeira* sp. (CAS, CASENT 9033287).

##### Remarks.

The lectotype is the larger of the two *Cambalida deminuta* males in the type series. Fernand Vaz is a river in the D.R. Congo and no specific locality is mentioned in the original description or on the label accompanying the types.

##### Diagnosis.

Females can be recognised by the broad spermathecae, small coiled lateral copulatory openings, and short entrance ducts of the female epigyne (Fig. 66). Males have a characteristically very short and narrow spiralling embolus (Figs 41, 68).

##### Female

**(Mayuka, CASENT 9033116).** Measurements: CL 2.05, CW 1.50, AL 2.55, AW 1.71, TL 4.53 (4.25–5.90), FL 0.13, SL 0.96, SW 0.90, AME–AME 0.06, AME–ALE 0.02, ALE–ALE 0.30, PME–PME 0.11, PME–PLE 0.06, PLE–PLE 0.49, PERW 0.70, MOQAW 0.27, MOQPW 0.40, MOQL 0.38.

Length of leg segments (sequence from femur to tarsus, and total): I 1.52 + 0.63 + 1.30 + 1.25 + 0.90 = 5.60; II 1.40 + 0.60 + 1.09 + 1.13 + 0.78 = 5.00; III 1.28 + 0.57 + 0.95 + 1.19 + 0.61 = 4.60; IV 1.75 + 0.63 + 1.48 + 1.78 + 0.80 = 6.44.

Carapace deep orange-brown, clypeus yellow-brown laterally, eye region slightly darker; black striae radiating from fovea towards palps and leg coxae; surface finely wrinkled, covered in white plumose setae. All eyes with black rings; AER procurved, ALE larger than AME; AME separated by distance slightly larger than ½ their diameter, AME separated from ALE by distance slightly less than ¼ AME diameter; clypeus height slightly less than 1⅓ AME diameter; PER procurved, PME very slightly smaller than PLE; PME separated by distance slightly less than ¾ their diameter, PME separated from PLE by distance slightly larger than ⅓ PME diameter; CW:PERW = 2.14:1. Chelicerae yellow-orange with black mottling on anterior surface, yellow along prolateral distal margin; three teeth on promargin, median tooth largest, proximal tooth smallest, distal tooth closer to median tooth than proximal tooth; two closely separated subequal teeth on retromargin, closer to fang base than promarginal teeth; endites yellow with black mottling, cream prolaterally; labium pale orange-brown with faint black mottling, cream distally; sternum orange with brown mottling, except at setal bases, giving speckled appearance. Legs finely granulate; femora I–IV dark brown, with paler dorsal line and pale retrolateral patch, extending to distal end ventrally; all femora yellow at distal end, also proximally on femora III and IV; patellae I–IV yellow with black mottling laterally; tibiae, metatarsi and tarsi I–III yellow with faint lateral black mottling; tibiae IV brown, yellow proximally and distally, with faint paired dorsal lines; metatarsi IV brown, yellow proximally and distally; tarsi IV yellow. Leg spination: femora: I pl 1 do 3, II do 3, III pl 1 do 3 rl 1, IV pl 1 do 3 rl 1; patellae with do 1 long distal seta; tibiae: I plv 1 rlv 1, II rlv 1, III pl 2 rl 2 plv 2 vt 2, IV pl 2 rl 2 plv 2 vt 2; metatarsi: I plv 2 rlv 2, II plv 2 rlv 2, III pl 2 rl 2 plv 1 rlv 1 vt 3, IV pl 3–4 rl 3 plv 2 rlv 1 vt 3. Palpal spination: femora do 2, patellae pl 1 do 1, tibiae pl 1 do 1 plv 1, tarsi pl 1 plv 3 rlv 1. Abdomen mottled dark grey dorsally; dorsal scutum orange-brown with black mottling, extending ¼ abdomen length; venter mottled pale grey, epigastric sclerite orange-brown, inframamillary sclerite yellow-brown. Epigyne with tiny lateral copulatory openings situated within small comma-shaped epigynal ridges (Fig. 66); copulatory ducts short and very narrow, curving obliquely, entering ST II posterolaterally; ST II large and oval, joined broadly to compact, broad, kidney-shaped posterior ST I (Fig. 67).

##### Male

**(lectotype, Fernand Vaz, MNHN 4109).** Measurements: CL 2.34, CW 1.68, AL 2.55, AW 1.55, TL 4.95 (TL 4.00–5.45), FL 0.23, SL 1.10, SW 1.03, AME–AME 0.09, AME–ALE 0.02, ALE–ALE 0.35, PME–PME 0.14, PME–PLE 0.07, PLE–PLE 0.50, PERW 0.77, MOQAW 0.34, MOQPW 0.47, MOQL 0.46.

Length of leg segments (sequence from femur to tarsus, and total): I 1.80 + 0.70 + 1.65 + 1.60 + 1.05 = 6.80; II 1.60 + 0.63 + 1.36 + 1.35 + 0.85 = 5.79; III 1.35 + 0.70 + 1.02 + 1.00 + 0.73 = 4.80; IV 2.20 + 0.75 + 1.95 + 2.35 + 1.00 = 8.25.

Carapace deep orange-brown with black mottling, clypeus slightly paler, eye region black; black striae radiating from fovea towards palps and leg coxae; surface finely wrinkled, densely covered in white plumose setae. All eyes with black rings; AER procurved, ALE much larger than AME; AME separated by distance slightly less than ⅔ their diameter, AME separated from ALE by distance slightly less than ^1^∕_5_ AME diameter; clypeus height slightly less than 1⅓ AME diameter; PER procurved, PLE slightly larger than PME; PME separated by distance equal to ¾ their diameter, PME separated from PLE by distance slightly less than ^2^∕_5_ PME diameter; CW:PERW = 2.18:1. Chelicerae orange-brown with faint black mottling on anterior surface, yellow-orange along prolateral distal margin; three teeth on promargin, median tooth largest, proximal and distal tooth smaller, subequal in size; distal tooth situated closest to median tooth; two slightly separated subequal teeth on retromargin, closer to fang base than promarginal teeth; endites yellow-brown, cream prolaterally; labium pale orange-brown proximally, cream distally; sternum pale orange-brown with black mottling. Legs finely granulate; femora I brown, yellow at distal end; femora II–IV brown in distal half, yellow-brown proximally and dorsally at distal end; patellae I–IV yellow with black mottling laterally, fused to faint black ring at distal end; tibiae I–III yellow dorsally and ventrally with faint black mottling laterally; tibiae IV brown, yellow at distal end; metatarsi I–IV yellow with faint black mottling in distal half; tarsi I–IV yellow. Leg spination: femora: I pl 1 do 3, II pl 1 do 3, III pl 2 do 3 rl 1, IV pl 2 do 3 rl 1; patellae with do 1 long distal seta; tibiae: I plv 1 rlv 1, II rlv 1, III pl 2 do 1 rl 2 plv 2 vt 2, IV pl 2 do 1 rl 2 plv 2 vt 2; metatarsi: I plv 2 rlv 2, II plv 2 rlv 2, III pl 2 rl 2 plv 1 rlv 1 vt 3, IV pl 3 rl 3 plv 2 rlv 2 vt 3. Palpal spination: femora pl 1 do 2, patellae pl 1, tibiae pl 1 plv 1, tarsi pl 1 plv 2. Abdomen with red-brown dorsal scutum with dense black mottling, nearly covering entire dorsum, with small white spot of dense plumose setae just above spinnerets; sides of abdomen mottled dark grey; venter mottled pale grey, epigastric sclerite, post-epigastric sclerites and ventral sclerite red-brown, inframamillary sclerite yellow-brown. Palps yellow-brown with faint black mottling; embolus very short, with one and a quarter narrow coils, tip directed distally (Figs 52, 68, 69).

##### Distribution.

Widespread in central and western Africa (Fig. 79).

##### Biology.

This species has been collected from a variety of tropical forest types, mainly by pitfall trapping, litter sifting and by hand.

#### Cambalida dippenaarae sp. n.

urn:lsid:zoobank.org:act:D1093D7A-46A6-403E-ACE4-09E66565DAFD

http://species-id.net/wiki/Cambalida_dippenaarae

Figures 1–4 , 14 [Fig F4] [Fig F5] –39, 42 , 44–49 , 53 , 70–73

##### Type material.

**Holotype male, together with one paratype male.** ZAMBIA: Livingstone, Quarry near Livingstone Airport, 17°47.998'S, 25°46.588'E, leg. C. Haddad & J. Parau, 1.XII.2006 (leaf litter) (NCA 2007/625).

##### Paratypes.

SOUTH AFRICA:*Eastern Cape Province*:Kei Mouth, 32°41.206'S, 28°22.497'E, leg. C. Haddad, 8.XII.2005 (leaf litter, coastal forest), 1♂ 2♀ (NCA 2006/1290). *Free State Province*: Bloemfontein, Free State National Botanical Gardens, 29°03'S, 26°13'E, leg. C. Haddad, 3.I.2011 (sifting leaf litter), 1♀ (NMBA 16157); Same locality, 29°02'S, 26°12'E, leg. V. Butler, 25.XI.2009 (*Cussonia paniculata* leaf litter), 1♂ (NMBA 15727); Same data, 18.IX.2009, 2♀ (NMBA 15666); Same locality, 29°08'S, 26°10'E, leg. L. Lotz, XI.2006 (pitfall traps, next to ridge under tree), 1♂ (NMBA 10941); Brandfort district, Florisbad Research Station, 28°46'S, 26°05'E, 1250m a.s.l., leg. L.N. Lotz, 1–15.II.1988 (pitfall traps), 1♀ (NMBA 3829). *KwaZulu-Natal Province*: Ndumo Game Reserve, Crocodile farm, 26°54.426'S, 32°19.185'E, leg. C. Haddad, 17.I.2006 (on ground surface), 1♂ 2♀ (NCA 2006/423); Same locality, Environmental Centre, 26°54.955'S, 32°18.376'E, leg. C. Haddad & V. Swart, 6.XII.2009 (base of grass tussocks, broadleaf woodland), 1♀ (TMSA 23628); Tembe Elephant Park, Sparse woodland, 26°57'S, 32°23'E, leg. C. Haddad, 6.I.2002 (searching, leaf litter), 1♀ (NCA 2007/3543); Same locality, 27°01'S, 32°24'E, leg. C. Haddad, 5.I.2002 (leaf litter, deep sand forest), 5♀ (NCA 2002/378); Umziki Pan, near Hluhluwe [28°02'S, 32°19'E], leg. P. Reavell, 12.II.1990 (at night on tree trunk), 1♀ (NMSA). *North-West Province*: Marikana, Buffelspoort, 25°45'S, 27°29'E, leg. A.S. Honiball, 30.XI.2006, 1♂ 1♀ (NCA 2007/1155); Vryburg district, Weltevrede Farm, 27°24.976'S, 24°29.906'E, leg. R. Lyle, R. Fourie, D. du Plessis & J. Adendorff, 8–12.I.2008 (garden, active collecting), 1♂ 2♀ (NCA 2009/3676). ZAMBIA: Wildlives Game Farm, near Choma, open savanna, 16°58.974'S, 26°38.974'E, leg. C. Haddad, 4.XII.2006 (leaf litter), 3♂ 7♀ (NCA 2007/552); Same locality, Nabuyani River, 16°59.615'S, 26°38.093'E, leg. C. Haddad, 3.XII.2006 (leaf litter), 7♂ 16♀ (NCA 2007/1126).

**Other material examined.** BOTSWANA: Okavango Delta, near Shakawe, Lesideng Research Camp, 18°25.822'S, 21°53.771'E, leg. C. Haddad, 25.XI.2006 (leaf litter), 1imm. 5♂ (NCA 2007/937); Same locality, leg. C. Haddad, 26–29.XI.2006 (night collecting), 1♂ 2♀ (NCA 2007/974); Same locality, leg. C. Haddad, 26.XI–11.XII.2006 (pitfalls, riverine forest), 1♀ (NCA 2007/1116); Samochima lagoon, Shakawe Fishing Camp, 18°25.749'S, 21°54.035'E, leg. C. Haddad, 10.XII.2006 (leaf litter), 1imm. 2♀ (NCA 2007/1050). MOZAMBIQUE: Morrungulo Resort, 12m a.s.l., 23°13.983'S, 35°29.587'E, leg. C. Haddad, R. Lyle & R. Fourie, 6.XII.2007 (leaf litter, dune forest), 2imm. 3♂ 1♀ (NCA 2008/188). NAMIBIA: Caprivi strip, 34km E of Divungu, 18°02.944'S, 21°54.611'E, leg. C. Haddad, 30.XI.2006 (under rocks), 2♀ (NCA 2007/907). SOUTH AFRICA: *Eastern Cape Province*: Cwebe Nature Reserve, The Haven, 32°14.497'S, 28°54.653'E, leg. C. Haddad, 30.X.2006 (grassy litter behind dunes), 1imm. 1♂ (NCA 2007/243); Great Fish River Reserve, at Selbourne, 33°08'S, 26°39'E, leg. M. Burger, 5.XII.1993 (pitfall traps), 1♀ (NCA 96/58); Sundays River Valley, 33°23'S, 25°26'E, leg. H. Potgieter, 23.XI.1999 (pitfall traps, citrus orchard), 4♂ (NCA 2000/236). *Gauteng Province*:Kloofendal Nature Reserve, near Roodepoort, 26°08'S, 27°52'E, leg. A. Leroy, 9.I.1988 (pitfall traps), 1♀ (NCA 89/151). *KwaZulu-Natal Province*: 15km N of Richard's Bay, 28°47'S, 32°06'E, leg. T. Wassenaar, 10.XII.1996 (pitfall traps, rehabilitated coastal forest), 1♂ (NCA 97/841); iSimangaliso [Greater St Lucia] Wetlands Park, Eastern Shores Nature Reserve, 29°05.726'S, 26°09.435'E, leg. C. Haddad, 3.VII.2007 (leaf litter), 1imm. 1♀ (NCA 2007/2899); iSimangaliso [Greater St Lucia] Wetlands Park, Hell's Gate, 28°02.3'S, 32°26.0'E, leg. J. Esterhuizen, 19.I.2003 (tsetse fly traps), 1♂ (NCA 2004/795); Ithala Game Reserve, Ngubhu loop, near ruins, 27°30.817'S, 31°14.304'E, leg. C. Haddad, R. Fourie & D. du Plessis, 1.VII.2007 (under rocks),6imm. 1♂ (NCA 2007/2814); Same locality, Doornkraal Camp, 27°30.735'S, 31°12.231'E, leg. C. Haddad & R. Fourie, 29.VI.2007 (sifting leaf litter), 14imm. 2♀ (NCA 2007/2875); Kosi Bay Nature Reserve, 26°57.767'S, 32°48.981'E, leg. C. Haddad, 15.IV.2006 (leaf litter, coastal forest), 1imm. 1♂ (NCA 2006/757); Ophathe Game Reserve, Montane grassland, 28°25.344'S, 31°23.957'E, 897m a.s.l., leg. C. Haddad, 4.X.2008 (sifting leaf litter), 6imm. 1♂ (NCA 2008/3900); Tembe Elephant Park, 27°01'S, 32°24'E, leg. C. Haddad, 5.I.2002 (leaf litter, deep sand forest), 2♂ 1♀ (NCA 2002/523). *Limpopo Province*: Klein Kariba, near Warmbaths [Bela-Bela], 24°52'S, 28°20'E, 1140m a.s.l., leg. A. Leroy, 27.XI.1996, 1♀ (NCA 2004/830); Little Leigh, 22°56.910'S, 29°52.177'E, leg. I. Sinthumule, 22.XI.2005 (gallery forest), 1♂ (NCA 2009/2041); Same locality, leg. B. van der Waal, 22.XI.2005 (gallery forest), 2♂ (NCA 2010/3324); Mabula Lodge, near Warmbaths [Bela-Bela], 24°50'S, 27°57'E, leg. J. Loubser, 16.VII.1989 (running with ants in leaf litter), 1♀ (NCA 91/431); Same locality, leg. J. Leroy, 16.XII.1989 (running with ants in leaf litter), 1♀ (NCA 91/434); Nylsvlei Nature Reserve, Naboomspruit, 24°39'S, 28°40'E, leg. G. Ferreira, 27.X.1975 (pitfalls), 1♂ (NCA 87/265); Roedtan, between Settlers and Tuinplaas (Springbokvlake), leg. M. van Jaarsveld, 26.III.2003 (pitfall traps, grass), 1♀ (NCA 2003/1336); Soutpansberg Mountains, Lajuma Mountain Retreat, Island 4, 23°01.894'S, 29°26.123'E, leg. M. Mafadza, 5.XII.2004 (woodland litter sifting), 1♂ 2♀ (NCA 2006/961); Same locality, Island 4, 23°01.894'S 29°26.123'E, leg. M. Mafadza, 28.XI.2004 (sifting leaf litter, sample 2), 1♀ (NCA 2005/1888); Same locality, leg. M. Mafadza, 28.XI.2004 (active searching), 1♂ (NCA 2005/2025); Same locality, leg. C. Haddad, 6.II.2008 (base of grass tussocks), 4♀ (NCA 2008/508); Tshulu, 22°35'S, 30°48'E, leg. S. Foord, 21.II.2008 (pitfall traps), 1♀ (NCA 2008/2876). *Mpumulanga Province*: Nelspruit, Agricultural College, 25°21'S, 31°46'E, leg. P. Stephen, 22.XII.1998 (pitfall traps, grapefruit orchard), 1♀ (NCA 99/133); Same locality, leg. P. Stephen, 12.XI.1999 (pitfall traps, citrus orchard), 2♂ 1♀ (NCA 2000/184). *North West Province*: Magaliesberg, Hartebeespoort, 25°43'S, 27°50'E, leg. A. Honiball, 10.XII.2008, 1♀ (NCA 2011/837). ZAMBIA: Kafue National Park, Near Namwala, Chibila Camp, 15°46.636'S, 26°00.405'E, leg. C. Haddad & J. Parau, 7.XII.2006 (leaf litter), 1♂ 1♀ (NCA 2007/572); Wildlives Game Farm, near Choma, Hunter's Camp, 16°58.957'S, 26°36.973'E, leg. C. Haddad, J. Parau & F. Jordaan, 3.XII.2006 (leaf litter), 1imm. 5♀ (NCA 2007/474); Same locality, Campsite, 17°03'S, 26°30'E, leg. F. Nyathi, 9–14.XII.1994 (drift fence pitfall trap), 1♂ (NMZ 11896). ZIMBABWE: Sengwa Wildlife Research Area, 18°10'S, 28°14'E, leg. M.S. Cumming, 15.I.2007, 1♂ (NCA 2007/1307).

**Figures 44–49.**
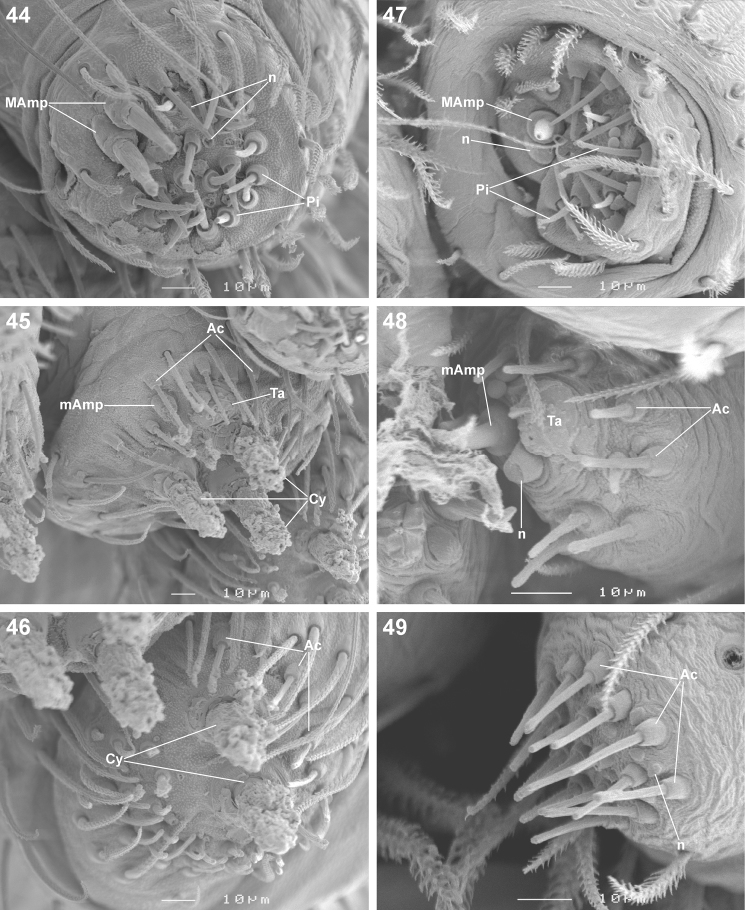
Scanning electron microscope photographs of *Cambalida dippenaarae* sp. n.female **(44–46)** and male **(47–49)** spinneret morphology: **44, 47** anterior lateral spinneret **45, 48** posterior median spinneret **46, 49** posterior lateral spinneret. Abbreviations: **Ac** aciniform gland spigot(s) **Cy** cylindrical gland spigot(s) **MAmp** major ampullate gland spigot(s) **mAmp** minor ampullate gland spigot(s) **n** nubbin(s) **Pi** piriform gland spigot(s) **ta** tartipore.

**Figures 50–56.**
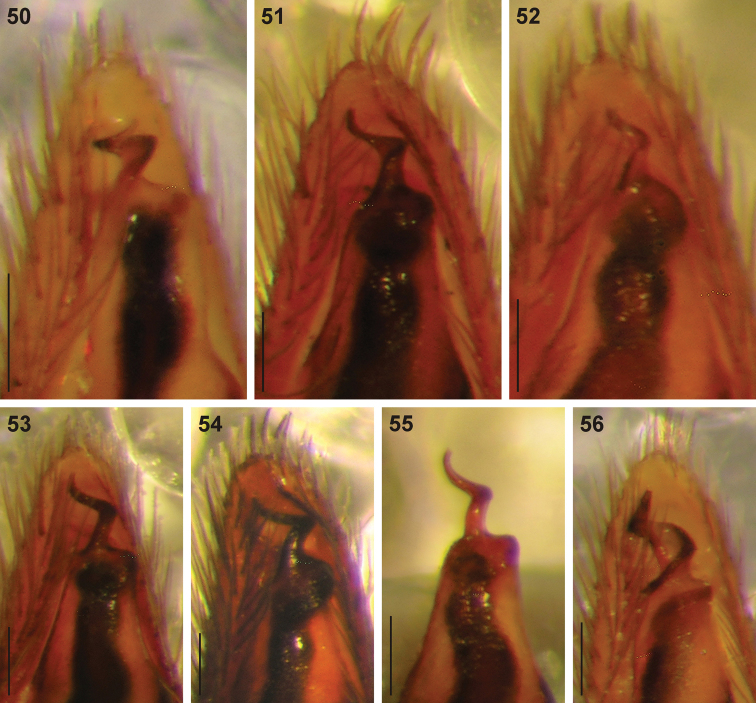
Digital microscope photographs of emboli of Afrotropical *Cambalida* species in ventral view: **50** *Cambalida compressa* sp. n. **51** *Cambalida coriacea* Simon, 1909 **52** *Cambalida deminuta* (Simon, 1909) **53** *Cambalida dippenaarae* sp. n. **54** *Cambalida fulvipes* (Simon, 1896) **55** *Cambalida griswoldi* sp. n. **56** *Cambalida loricifera* (Simon, 1885). Scale bars = 0.1mm.

**Figures 57–60.**
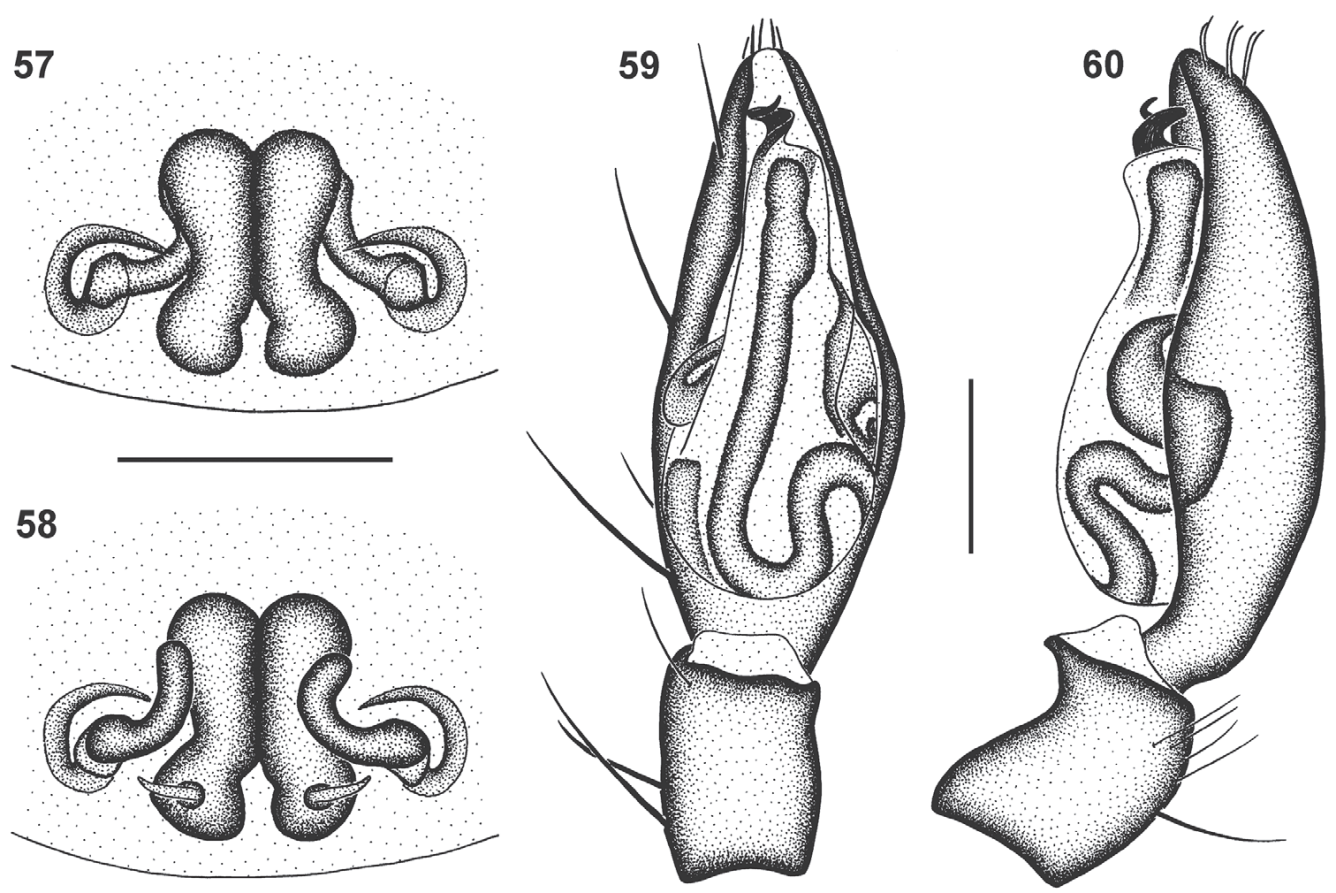
Genitalic morphology of *Cambalida compressa* sp. n.: **57** female epigyne, ventral view **58** same, dorsal view **59** male palp, ventral view **60** same, retrolateral view. Scale bars = 0.25mm.

**Figures 61–64.**
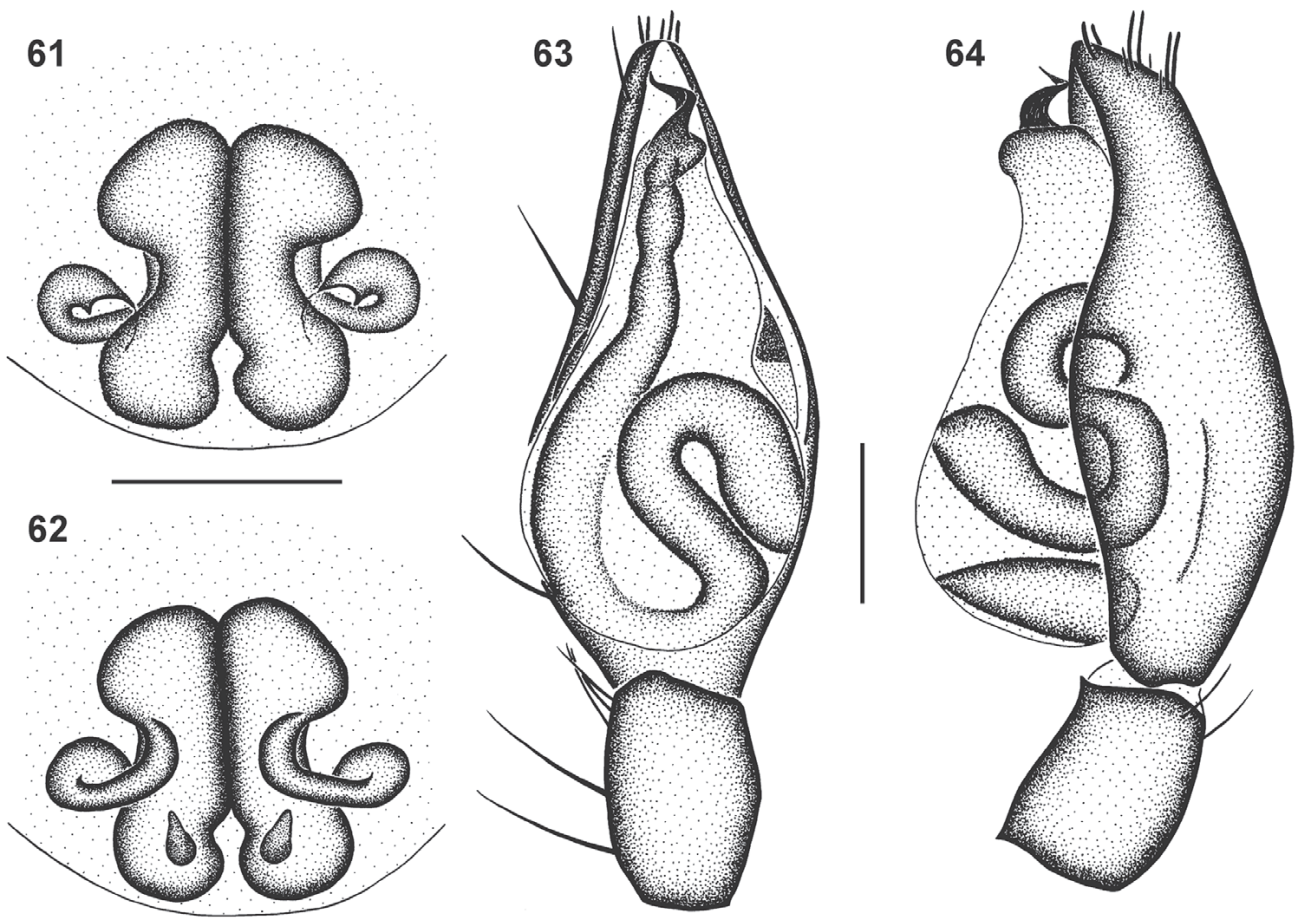
Genitalic morphology of *Cambalida coriacea* Simon, 1909: **61** female epigyne, ventral view **62** same, dorsal view **63** male palp, ventral view **64** same, retrolateral view. Scale bars = 0.25mm.

**Figure 65.**
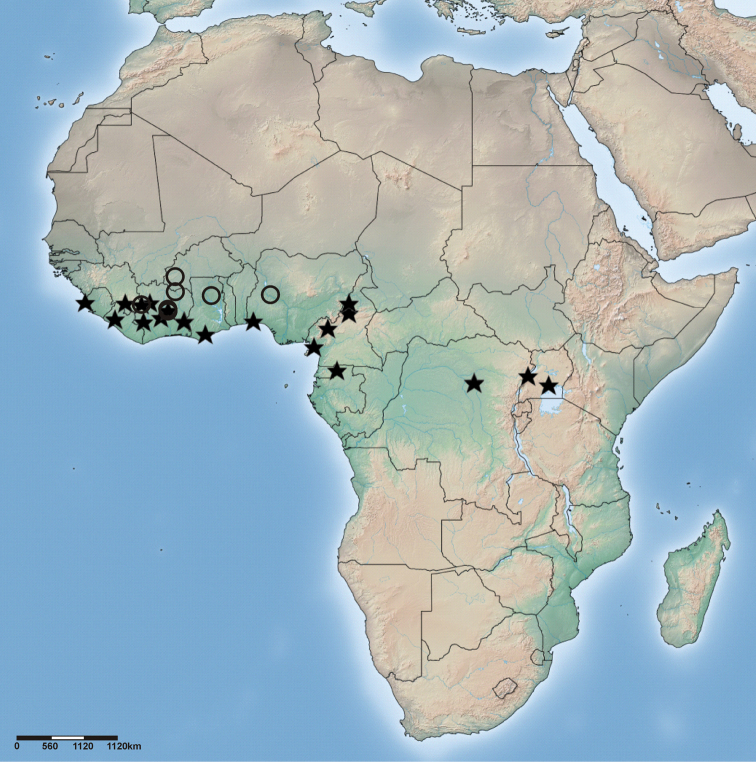
Distribution of *Cambalida compressa* sp. n. (open circles) and *Cambalida coriacea* Simon, 1909 (stars) in the Afrotropical Region.

**Figures 66–69.**
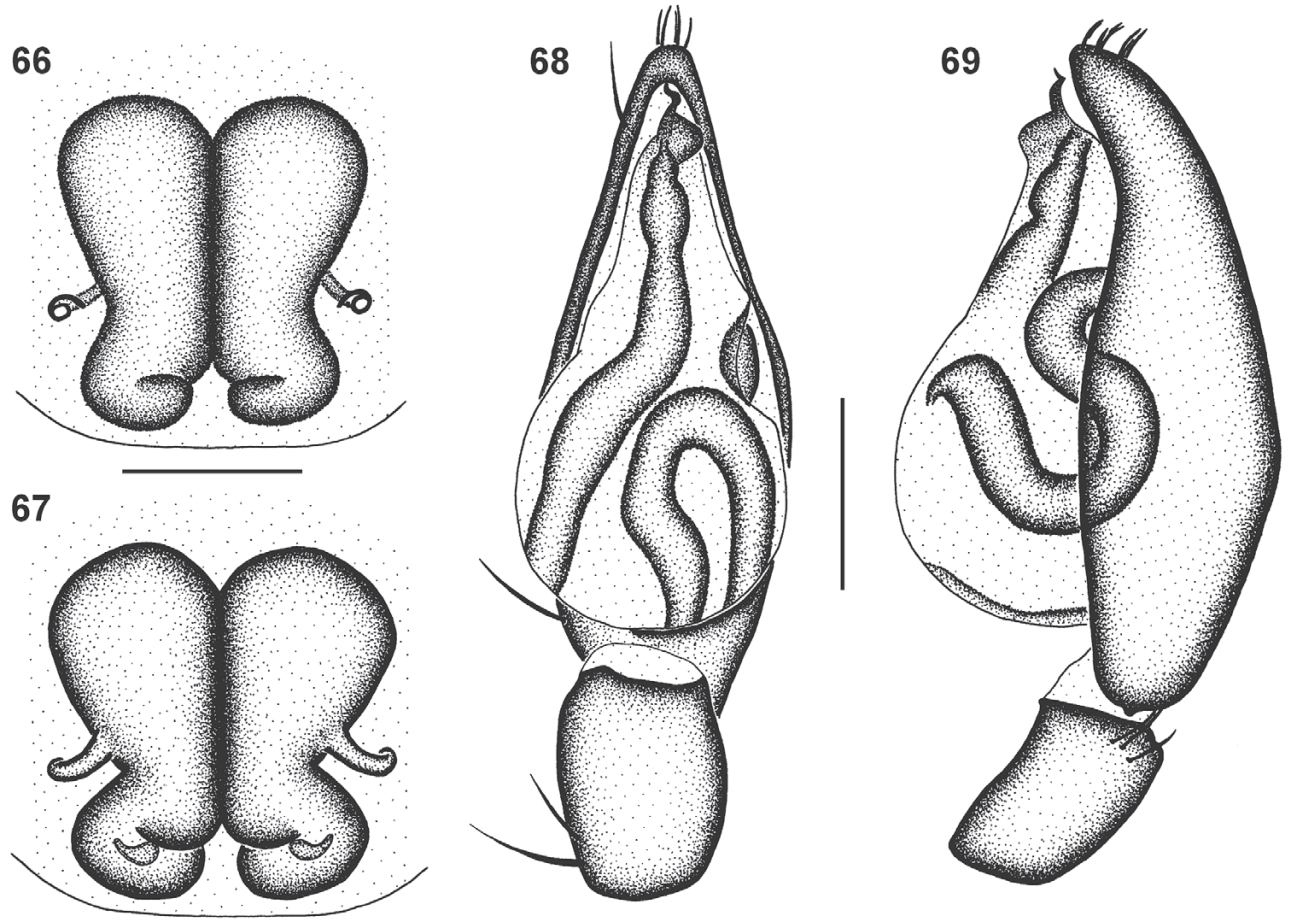
Genitalic morphology of *Cambalida deminuta* (Simon, 1909): **66** female epigyne, ventral view **67** same, dorsal view **68** male palp, ventral view **69** same, retrolateral view. Scale bars = 0.25mm.

**Figures 70–73.**
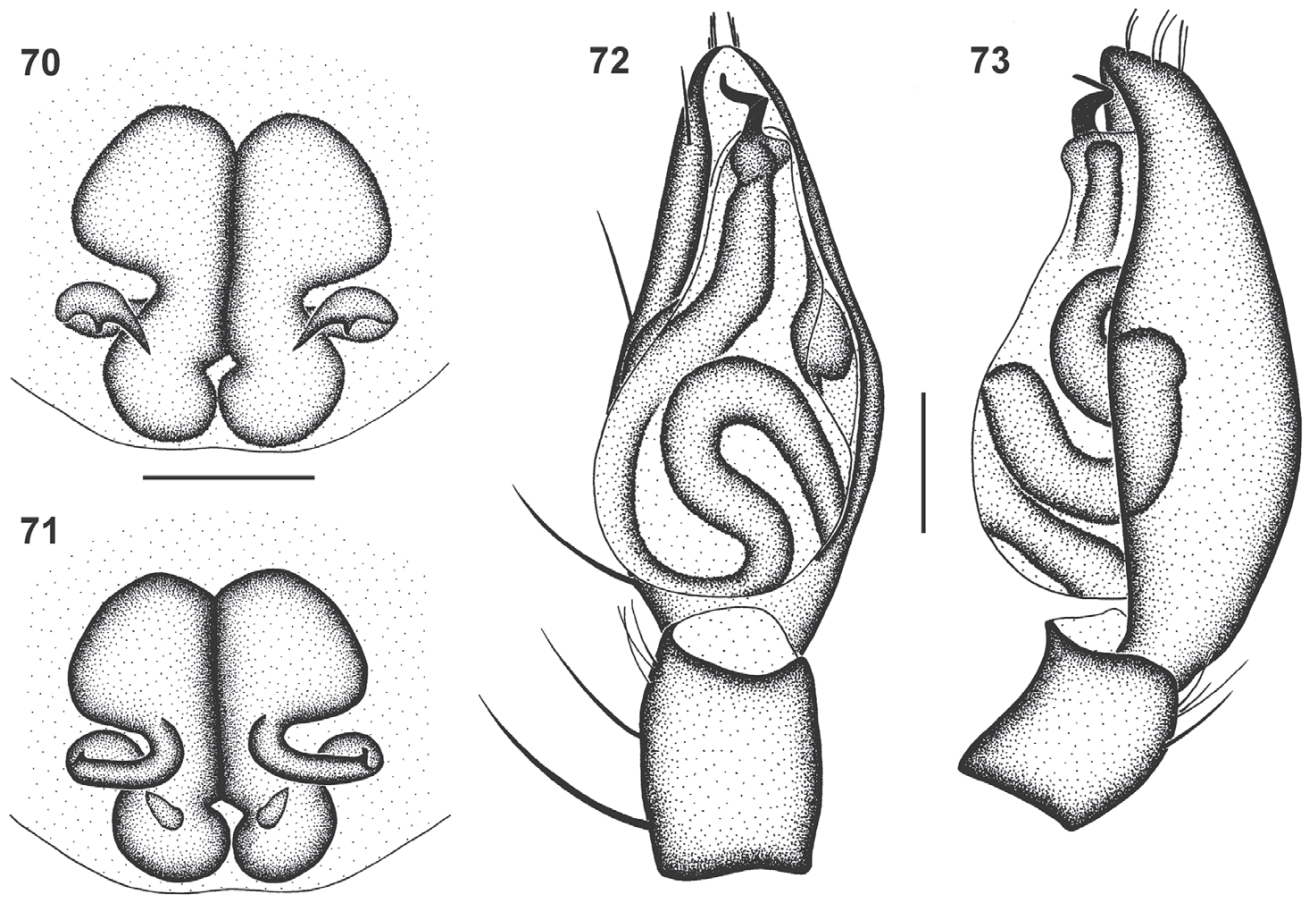
Genitalic morphology of *Cambalida dippenaarae* sp. n.: **70** female epigyne, ventral view **71** same, dorsal view **72** male palp, ventral view **73** same, retrolateral view. Scale bars = 0.25mm.

##### Diagnosis.

Females are closely related to *Cambalida fulvipes* but can be recognised by the subtriangular rather than round ST II and the narrower epigynal ridges (compare Fig. 70 with Fig. 80). Males can be recognised by the nearly parallel-sided basal section and the narrowly coiled distal section of the embolus.

##### Etymology.

The species name is a patronym in honour of Ansie Dippenaar-Schoeman, in recognition of her contributions to the study and promotion of research on African arachnids.

##### Female

**(holotype, Livingstone, NCA 2007/625).** Measurements: CL 2.53, CW 1.85, AL 3.15, AW 2.07, TL 5.75 (4.90–5.95), FL 0.22, SL 1.16, SW 1.11, AME–AME 0.06, AME–ALE 0.02, ALE–ALE 0.35, PME–PME 0.11, PME–PLE 0.04, PLE–PLE 0.53, PERW 0.83, MOQAW 0.33, MOQPW 0.49, MOQL 0.48.

Length of leg segments (sequence from femur to tarsus, and total): I 1.78 + 0.80 + 1.50 + 1.40 + 0.95 = 6.43; II 1.63 + 0.74 + 1.28 + 1.25 + 0.84 = 5.74; III 1.48 + 0.71 + 1.13 + 1.36 + 0.68 = 5.36; IV 2.18 + 0.88 + 2.03 + 2.28 + 0.92 = 8.29.

Carapace deep red-brown with black mottling, clypeus dark brown medially, yellow-brown laterally, eye region nearly black; faint black striae radiating from fovea towards palps and leg coxae; surface granulate, sparsely covered in white plumose setae. All eyes with black rings; AER procurved, ALE much larger than AME; AME separated by distance slightly less than ½ their diameter, AME separated from ALE by ^1^∕_6_ AME diameter; clypeus height slightly larger than AME diameter; PER procurved, PLE very slightly larger than PME; PME separated by distance slightly less than ^3^∕_5_ their diameter, PME separated from PLE by distance equal to ^1^∕_5_ PLE diameter; CW:PERW = 2.23:1. Chelicerae dark orange-brown, orange proximally and along prolateral distal margin; three teeth on promargin, median tooth largest, distal tooth slightly smaller than proximal tooth, distal tooth situated closest to median tooth; two teeth on retromargin, distal tooth slightly larger than proximal tooth, closer to fang base than promarginal teeth; endites dark brown, fading to yellow and cream prolaterally; labium dark brown, creamy-yellow distally; sternum deep orange-brown with dark brown mottling. Legs finely granulate; femora I–IV dark brown, with slightly paler lines dorsally, bright yellow dorsally at distal end; patellae I–III yellow with faint black mottling ventrally, black around patellar indentation; patellae IV yellow-orange with faint black mottling laterally and ventrally, black around patellar indentation; tibiae, metatarsi and tarsi I–III bright yellow-orange; tibiae IV orange with dense black mottling, except proximally and distally; metatarsi IV orange with faint black mottling; tarsi IV yellow with faint black mottling. Leg spination: femora: I pl 1 do 3, II do 3, III pl 1 do 3 rl 1, IV pl 1 do 3 rl 1; patellae with do 1 long distal seta; tibiae: I and II spineless, III pl 2 rl 2 plv 2 vt 2, IV pl 2 do 1 rl 2 plv 2 vt 2; metatarsi: I plv 2 rlv 2, II plv 2 rlv 2, III pl 2 rl 2 plv 2 rlv 2 vt 3, IV pl 3 rl 3 plv 2 rlv 2 vt 3. Palpal spination: femora do 2, patellae pl 1 spine do 2 setae, tibiae pl 1 do 1 plv 1, tarsi pl 1 plv 3 rlv 1. Abdomen dark grey, with dark red-brown dorsal scutum extending slightly more than ¼ abdomen length, with fine cream chevrons posteriorly and small white spot of dense plumose setae just above spinnerets; venter mottled creamy-grey, slightly darker towards spinnerets; epigastric scutum bright yellow-orange with black mottling, inframamillary sclerite yellow-brown. Epigyne with lateral copulatory openings situated within small curved epigynal ridges (Fig. 70); copulatory ducts initially directed anterolaterally, looping posteriorly, then transversely and anteriorly, entering ST II posteromedially; ST II somewhat triangular, with sharply angled lateral margins, joined narrowly to kidney-shaped posterior ST I (Fig. 71).

##### Male

**(paratype, Livingstone, NCA 2007/625).** Measurements: CL 2.40, CW 1.75, AL 2.90, AW 1.48, TL 5.45 (4.47–5.45), FL 0.19, SL 1.11, SW 0.99, AME–AME 0.06, AME–ALE 0.02, ALE–ALE 0.31, PME–PME 0.10, PME–PLE 0.06, PLE–PLE 0.51, PERW 0.76, MOQAW 0.30, MOQPW 0.43, MOQL 0.44.

Length of leg segments (sequence from femur to tarsus, and total): I 1.85 + 0.70 + 1.65 + 1.60 + 1.10 = 6.90; II 1.64 + 0.68 + 1.30 + 1.40 + 0.90 = 5.92; III 1.52 + 0.66 + 1.15 + 1.45 + 0.77 = 5.55; IV 2.30 + 0.83 + 2.11 + 2.55 + 1.06 = 8.85.

Carapace dark red-brown with black mottling, clypeus slightly paler laterally, eye region darker; faint black striae radiating from fovea towards palps and leg coxae; surface finely granulate, densely covered in white plumose setae. All eyes with black rings; AER procurved, ALE much larger than AME; AME separated by distance slightly less than ½ their diameter, AME separated from ALE by distance equal to ⅛ AME diameter; clypeus height slightly larger than 1½ AME diameter; PER procurved, PLE very slightly larger than PME; PME separated by distance slightly more than ^3^∕_5_ their diameter, PME separated from PLE by distance equal to ⅓ PME diameter; CW:PERW = 2.30:1. Chelicerae brown with black mottling on anterior surface, paler proximally, yellow along prolateral distal margin; three teeth on promargin, median tooth largest, distal tooth smaller than proximal tooth, distal tooth situated closest to median tooth; two slightly separated subequal teeth on retromargin, closer to fang base than promarginal teeth; endites dark yellow-brown with dark brown mottling, fading to yellow and cream prolaterally; labium orange-brown proximally, creamy-yellow distally; sternum deep orange-brown with dense black mottling. Legs finely granulate; femora I and II pale yellow-brown, yellow distally; femora III and IV dark orange-brown, yellow distally; patellae I and II yellow, and III and IV yellow-orange, all with faint black lateral mottling, black around patellar indentation; tibiae, metatarsi and tarsi I and II yellow with faint black mottling laterally; tibiae, metatarsi and tarsi III yellow-orange with faint black mottling laterally; tibiae IV deep orange with black mottling, yellow distally; metatarsi IV yellow-orange with black mottling, absent proximally and distally; tarsi IV yellow. Leg spination: femora: I pl 1 do 3, II do 3, III pl 1 do 3 rl 1, IV pl 1 do 3 rl 1; patellae with do 1 long distal seta; tibiae: I plv 0-1, II rlv 0-1, III pl 2 rl 2 plv 2 vt 2, IV pl 2 do 1 rl 2 plv 2 vt 2; metatarsi: I plv 2 rlv 2, II plv 2 rlv 2, III pl 2 rl 2 plv 1 rlv 1 vt 3, IV pl 3 rl 3 plv 2 rlv 2 vt 3. Palpal spination: femora do 2, patellae pl 1 spine do 2 setae, tibiae pl 1 plv 1, tarsi pl 2 plv 2. Abdomen with deep red-brown, nearly black, dorsal scutum covering entire dorsum, with small white spot of dense plumose setae just above spinnerets; sides of abdomen mottled dark grey; venter pale grey, epigastric sclerite, post-epigastric sclerites, ventral sclerite and inframamillary sclerite red-brown with black mottling. Palps yellow-brown with black mottling, cymbium orange-brown; embolus with parallel-sided basal section, distal section of coil transverse, bent at right angle to distally-directed tip (Figs 53, 72); embolic coil relatively narrow (Fig. 73).

##### Distribution.

Widespread throughout southern Africa (Fig. 79); largely sympatric with *Cambalida fulvipes* across its range (Fig. 95).

##### Biology.

A fairly common ground-dwelling spider collected mainly by litter sifting and pitfall traps in forest and savanna habitats. Occasionally collected from the Grassland Biome in South Africa.

#### Cambalida fagei

(Caporiacco, 1939)

http://species-id.net/wiki/Cambalida_fagei

Figures 74 –78

Brachyphaea fagei Caporiacco, 1939: 356, fig. 17 comb. n.

##### Type material.

**Holotype female.** ETHIOPIA: Neghelli [Negele, 05°19'N, 39°35'E], leg. E. Zavattari, 30.III.1937, deposited in MZUF, type no. 118, mag no. 876 (examined).

##### Diagnosis.

Females of this species share with *Cambalida deminuta* the very large ST II and compact ST I, but can be distinguished from that species by the much larger copulatory openings (Figs 77, 78).

##### Remarks.

**Figures 74–76.**
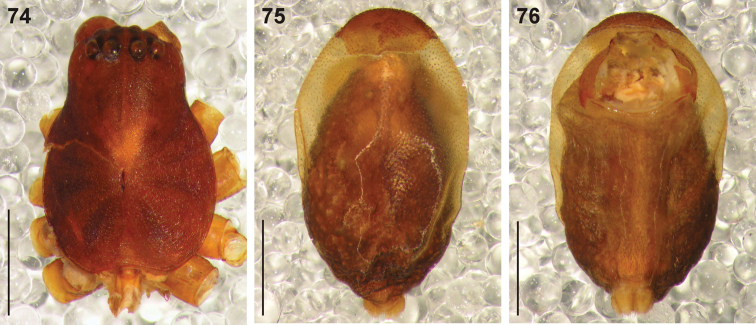
Digital microscope photographs of the holotype female of *Cambalida fagei* (Caporiacco, 1939): **74** carapace, dorsal view **75** abdomen, dorsal view **76** same, ventral view. Scale bars: 1.0mm.

**Figures 77–78.**
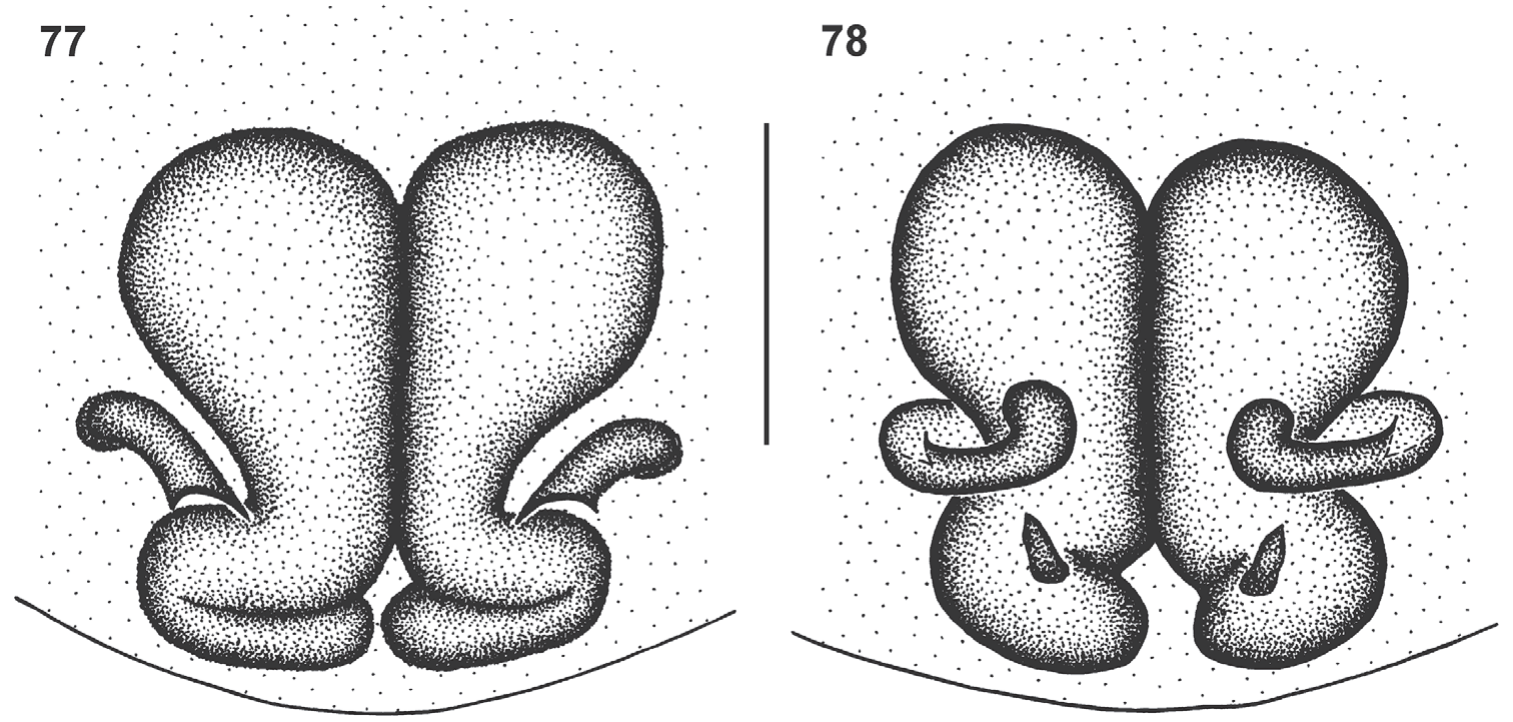
Genitalic morphology of *Cambalida fagei* (Caporiacco, 1939): **77** female epigyne, ventral view **78** same, dorsal view. Scale bar = 0.25mm.

The holotype female is in very poor condition but the eye arrangement (Fig. 74) and abdominal sclerotisation (Figs 75, 76) are consistent with the placement of this species in *Cambalida*. No material other than the holotype could be found and it will not be redescribed here. The epigyne of the species is distinct, notably the very large ST II and compact ST I (Figs 77, 78), and it is clearly different to *Cambalida fulvipes*, which also occurs in Ethiopia.

##### Distribution.

**Figure 79.**
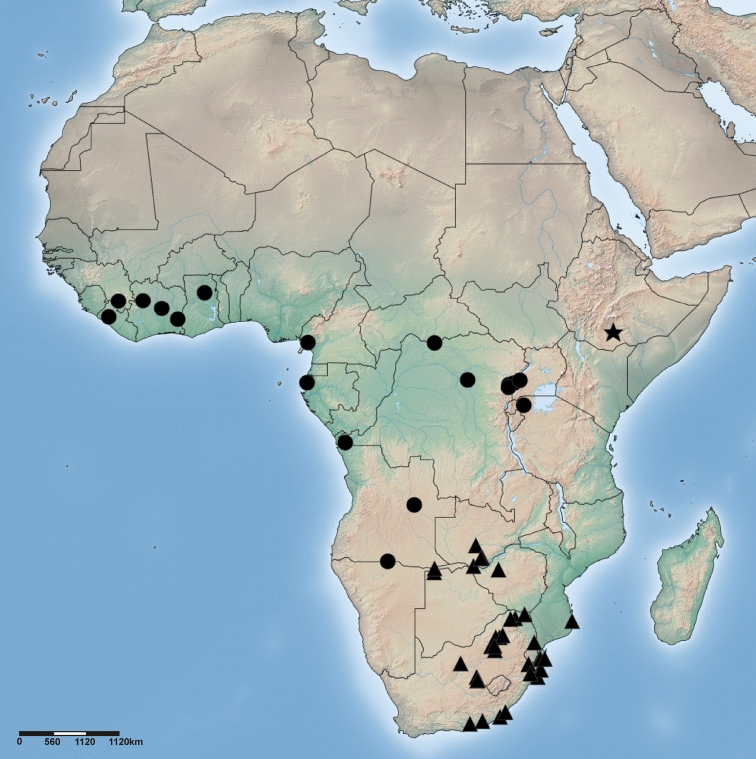
Distribution of *Cambalida deminuta* (Simon, 1909) (circles), *Cambalida dippenaarae* sp. n. (triangles) and *Cambalida fagei* (Caporiacco, 1939) (star) in the Afrotropical Region.

Known from the type locality only (Fig. 79).

##### Biology.

Unknown.

#### Cambalida fulvipes

(Simon, 1896)

http://species-id.net/wiki/Cambalida_fulvipes

Figures 5, 6, 8–13 , 54 , 80–83

Castianeira fulvipes Simon, 1896: 406 comb. n.
Castianeira depygata Strand, 1916: 91 syn. n.
Castianeira mestrali Lessert, 1921: 424 syn. n.

##### Type material.

**Lectotype female and paralectotype female, here designated.** SOUTH AFRICA: Pretoria [25°42'S, 28°13'E], MNHN 18324 (examined).

##### Type material of synonyms.

*Castianeira depygata* Strand, 1916. Holotype male. D.R. CONGO: Lake Kivu, Kwidjwi Island [Idjwi Island, 02°10'S, 29°03'E], leg. A.F. Herzog, IX.1907, ZMB 27150 (examined); *Castianeira mestrali* Lessert, 1921. Lectotype male and paralectotype female, here designated. TANZANIA: Kibonoto [Kibongoto, 03°11'S, 37°06'E], IX–X.1905? (zone des cultures), MNHG (examined).

##### Other material examined.

BOTSWANA: Maphaneng Pan, near Maun [19°56'S, 23°25'E], leg. A. Russell-Smith, 13.XI.1976 (mopane woodland), 1♀ (PCRS); Okavango Delta, Pom Pom, 19°35.072'S, 22°50.560'E, leg. E. Kassimatis, 17.VIII.2001 (sweeping, pitfalls), 1imm. 1♂ (NCA 2007/1314); Okavango Delta, Shakawe Fishing Camp, 18°26'05"S, 20°54'23"E, leg. J. van As, 26.IV–7.V.2005 (pitfall traps, forest), 2♀ (NCA 2006/819); Okavango Delta, Xugana island, 130km NNE of Maun, 19°04'S, 23°03'E, leg. B. Lamoral, 18–21.XI.1980, 1♀ (NMSA 20265), 1♂ (NMSA 20266), 1♀ (NMSA 20267), 1♀ (NMSA 20270); Same data, 19–24.XI.1980, 1♀ (NMSA 22011); Same data, 21–22.XI.1980, 1♀ (NMSA 22012). CAMEROON: Faro Game Reserve, 08°24'N, 12°49'E, leg. R. Jocqué, K. Loosveldt, L. Baert & M. Alderweireldt, 5.V.2007 (gallery forest, pitfall), 1♂ (MRAC 221185); Same locality, leg. R. Jocqué, K. Loosveldt, L. Baert & M. Alderweireldt, 5.V.2007 (mature gallery forest, pitfall), 1♂ (MRAC 221214); Same locality, leg. R. Jocqué, K. Loosveldt, L. Baert & M. Alderweireldt, 29.IV.2007 (termite mound), 1♂ (MRAC 221372); Mabete [Mabeta, 04°00'N, 09°17'E], leg. B. Malkin, 24.V–7.VI.1949, 3♂ 1♀ (CAS, CASENT 9033127); Mount Cameroun, near Buea, 04°12'N, 09°11'E, 1200m a.s.l., leg. Bosmans & Van Stalle, 12.III.1981 (meadow), 2♂ (MRAC 162109); Muyuka [04°17'N, 09°24'E], leg. B. Malkin, 24–29.VI.1949, 1♂, together with 1♀ *Cambalida deminuta* (CAS, CASENT 9033116). D.R. CONGO: Fernand Vaz River, 1♂, together with lectotype and paralectotype of *Cambalida deminuta* (MNHN 4109). *Kivu*: Ruindi Plain, leg. M. Lejeune, 10.VII.1972 (battage), 2imm. 1♀ (MRAC 144623); Ruzizi Plain [02°55'S, 29°04'E], Gikanga sector, 890m a.s.l., leg. S. Ndani, V.1966, 2♂ 2♀ (MRAC 130608), 1♂ (MRAC 130609); Same locality, 900m a.s.l., leg. S. Ndani, V.1966 (dans terreau de bamboos), 2♂ 3♀ (MRAC 130586). ETHIOPIA: Abernosa Ranch, Near Adami Tulu, 07°52'N, 38°42'E, 1600m a.s.l., 23.V.1981, leg. A. Russell-Smith (under log, *Acacia tortilis* woodland), 1♂ (PCRS); Addis Ababa, 09°00'N, 38°45'E, 27.IX.1982, leg. A. Russell-Smith (in grazed grassland, course of Bote stream), 1♂ (PCRS); Awash National Park, Compound of Ras Hotel, 09°05'N, 40°00'E, 22.VI.1983, leg. A. Russell-Smith (in heap of cut grass), 1♂ (PCRS); Moyale, 03°33'N, 39°03'E, leg. W.J. Pulawski, 10.VII.1997, 1♀ (CAS, CASENT 9033098); 10 km E of Addis Ababa, Road to Debre Berhan, 09°02'N, 38°14'E, 2400m a.s.l., 15.VI.1987, leg. A. Russell-Smith (litter of semi-deciduous montane scrub), 1♂ 1♀ (PCRS). GABON: *Estuaire*: Ntoum, 00°23'N, 09°47'E, leg. A. Pauly, VII–IX.1985 (milieux divers), 1♂ (MRAC 172996); Same locality, leg. A. Pauly, 7.XI.1985 (carrière de sable, piège bac d'eau), 1♂ (MRAC 172933); Kango, bords du Komo, 00°09'N, 10°08'E, leg. A. Pauly, 17.XI.1985 (piège bac d'eau), 1♀ (MRAC 173037). IVORY COAST: Appouesso, 06°35'N, 03°28'W, leg. R. Jocqué, 20.XI.1994 (cocoa plantation, W of village), 1♂ (MRAC 201033); Same locality, leg. R. Jocqué, 21.XI.1995 (in house), 1♂ (MRAC 202685); Bandama River, N of Korhogo, 09°27'N, 05°38'W, leg. J. Everts, V.1980 (centre riverine forest), 1♂ (MRAC 172291); Same data, VI.1980, 1♂ (MRAC 172288); Bientotkro, near Gagnoa [06°07'N, 05°56'W], leg. A. RussellSmith, 27.VIII.1992 (in valley rice field), 1♂ 6♀ (PCRS); Bouaké [07°41'N, 05°02'W], leg. A. Russell-Smith, 21.VIII.1994 (under stones, rocky outcrop in degraded savanna), 1♀ (PCRS); Same locality, West African Rice Development Association, leg. A. Russell-Smith, 17.VIII.1994 (weed control experiment), 3♂ 10♀ (PCRS); Dobouo [06°51'N, 06°32'E], leg. A. Russell-Smith, 26.VIII.1992 (in harvested upland rice field), 2♂ 4♀ (PCRS); Gagnoa [06°07'N, 05°56'W], leg. A. Russell-Smith, 14.III.1995 (pitfall, upland rice), 23♂ 14♀ (PCRS); Same locality, leg. A. Russell-Smith, 5.VIII.1995 (in upland rice after harvest), 2♂ 2♀ (PCRS); Ganhoué [08°11'N, 07°51'W], leg. A. Russell-Smith, 27.VIII.1987 (in upland rice field), 1♀ (PCRS); Mankono, Ranch de la Marahoué, 08°27'N, 06°52'W, leg. J. Everts, II.1980 (riverine forest), 1♀ (MRAC 172277); Same data, III.1980, 3♀ (MRAC 172269); Same data, IV.1980, 2♂ (MRAC 172270); Same data, V.1980, 3♀ (MRAC 172266); Odienné, Idessa station [09°30'N, 07°34'W], leg. A. Russell-Smith, 20.VIII.1992 (in upland rice field), 1♀ (PCRS); Serifoula [08°07'N, 07°57'W], leg. A. Russell-Smith, 21.VIII.1993 (in harvested upland rice field), 1♀ (PCRS); Taï Forest, Ecological Research Centre, 05°50'N, 07°21'W, leg. R. Jocqué & D. Van den Spiegel, 22.II.2010 (under planks in base camp, hand catch), 1♀ (MRAC 233650). KENYA: Amboseli National Park [02°40'S, 37°15'E], A. Russell-Smith, 7.I.1985 (in *Acacia* woodland), 3♀ (PCRS); Homa Bay Farmers Training Centre, 00°27'S, 34°24'E, leg. C. Midega, 15.III.2004 (pitfall traps, maize fields), 1♀ (NCA 2004/1340), 1♂ (NCA 2004/1343), 1♂ (NCA 2004/1358), 1♂ 1♀ (NCA 2006/1351), 1♂ (NCA 2004/1348), 1♀ (NCA 2004/2132); Mount Kasigau, Jora village, 03°50'S, 38°39'E, leg. E. Selempo, XII.2001 (pitfall trap), 1♂ (MRAC 213091); Nairobi, Garden at Muthaiga [01°15'S, 36°50'E], leg. A. Russell-Smith, 30.XII.1977 (in short grass and under stones), 2♀ (PCRS); Ngaia Forest, 00°19'N, 38°02'E, leg. R. Jocqué, C. Warui & D. Van den Spiegel, 24.IV.2004 (sieved litter), 1♀ (MRAC 215238); Same locality, 1071m a.s.l., leg. D. Van den Spiegel, 3.XII.2002, 1♀ (MRAC 220167). MALAWI: Chintheche, 11°50'S, 33°13'E, leg. R. Jocqué, II.1977, 1♂ (MRAC 152368); Michiru Wildlife Reserve, near Blantyre, 15°45'S, 34°58'E, leg. A. Russell-Smith, 27.X.1996, 1♀ (PCRS). MOZAMBIQUE: Inhaca Island, 26°01'S, 32°54'E, leg. T. Steyn, 28.V–19.VI.1994 (beach and dunes, by hand), 2♂ (MRAC 215980); Same locality, leg. T. Steyn, 5–19.II.1994 (coastal woodland, pitfalls), 1♂ 1♀ (MRAC 208942); Same data, 2–16.X.1993, 1♀ (MRAC 209033); Same data, 30.X–13.XI.1993, 1♂ (MRAC 209057); Same data, 13–27.XI.1993, 3♂ 1♀ (MRAC 209294); Same data, 5–19.III.1994, 1♀ (MRAC 209437); Same data, 25.VI–9.VII.1994, 1♀ (MRAC 209464); Same data, 19.III–2.IV.1994, 1♂ (MRAC 209731); Same data, 14–28.V.1994, 2♂ (MRAC 209773); Same data, 23.VII–6.VIII.1994, 4♂ 1♀ (MRAC 209878); Same data, 19.II–5.III.1994, 1♀ (MRAC 209893); Same locality, leg. T. Steyn, 11–25.XII.1993 (open parkland, pitfalls), 1♂ (MRAC 209687); Same locality, leg. T. Steyn, 4–18.X.1993 (wetland, pitfalls), 1♂ (MRAC 209068); Same data, 28.V–19.VI.1994, 1♀ (MRAC 209718); Same data, 25.VI–9.VII.1994, 1♂ (MRAC 208987). NIGERIA: *Western State*: Ibadan, I.I.T.A., 07°14'N, 03°30'E, leg. A. Russell-Smith, 7.VI.1981 (short grass beside lake), 1♀ (MRAC 177278). RWANDA: Butare, 02°36'S, 29°44'E, leg. P. Nyalugaka, VI–VII.1971, 1♂ 1♀ (MRAC 140729). SOUTH AFRICA: *Eastern Cape Province*: Mpofu Nature Reserve, 32°36'S, 26°36'E, leg. S. Peinke (in building), 1♀ (NCA 2011/823); St Francis Bay, 34°08'S, 24°50'E, leg. A. Leroy, 18.XII.2006 (in leaf litter, under vegetation, static dunes), 1♂ (NCA 2008/1989); W of Sterkstroom, Bamboesberg, Farm Bamboeshoek, 31°36'S, 26°22'E, leg. M. Burger, M. Fabricius & C. Lakoy, 1♀ (NCA 2008/1895). *Free State Province*: Bloemfontein, 29°08'S, 26°10'E, leg. S. Louw, 8.XII.1988 (in house), 1♀ (NMBA 2800); Bloemfontein, Bain's Vlei, 29°02.764'S, 26°04.780'E, leg. V. Swart, 26.XII.2011 (on floor in- side house), 1♂ (TMSA 24131); Bloemfontein, Free State National Botanical Gardens, 29°08'S, 26°10'E, leg. R. Poller & S. Otto, XII.2006 (pitfall traps), 2♂ (NMBA 11165); Same locality, 29°02'S, 26°12'E, leg. V. Butler, 18.IX.2009 (*Searsia lancea* leaf litter), 1♀ (NMBA 15672); Same locality, 29°03'S, 26°13'E, leg. C. Haddad, X.2010 (base of grass tussocks), 1♀ (NMBA 16163); Bloemfontein district, Farm Deelhoek, 28°51'S, 26°07'E, leg. C. Haddad, 17.XI.2001 (*Eucalyptus* leaf litter), 1♂ 2♀ (NCA 2002/500); Bloemfontein district, Hopefield farm, 28°54'S, 26°14'E, leg. C. Haddad, 28.X.2001 (bluegum leaf debris), 1imm. 3♂ 1♀ (MRAC 214919); Same locality, leg. C. Haddad, 18.XI.2001 (*Eucalyptus* leaf litter), 5♀ (NCA 2002/501); Same locality, leg. C. Haddad, 22.XII.2002 (kukuyu grass along reservoir), 1♀ (NCA 2002/502); Brandfort district, Florisbad Research Station, 28°46'S, 26°05'E, 1250m a.s.l., leg. Museum Staff, III.1983 (pitfall traps), 1♀ (NMBA 351); Same locality, leg. L.N. Lotz, 23.XI–8.XII.1987 (pitfall traps), 1♂ (NMBA 8315), 1♂ (NMBA 8459); Same locality, leg. L.N. Lotz, 8–21.XII.1987 (pitfall traps), 1♂ (NMBA 8464), 1♂ (NMBA 9331); Same locality, leg. L.N. Lotz, 31.X–18.XI.1988 (pitfall traps), 1♂ (NMBA 4814), 1♂ (NMBA 4884), 1♂ (NMBA 4909), 1♂ (NMBA 4919); Erfenis Dam Nature Reserve, 28°30'S, 26°48'E, leg. C. Haddad, S. Otto & R. Poller, 22.X–22.XI.2005 (pitfall traps, unburned site 2), 1♂ (NMBA 13943); Ladybrand district, Farm De Luc, 29°17.524'S, 27°24.267'E, leg. C. Haddad, 5.XII.2008 (base of grass tussocks), 2♀ (NCA 2008/4282); Sandveld Nature Reserve, 27°41'S, 25°43'E, leg. C. Haddad, 22.IX.2003 (*Acacia erioloba* leaf litter), 2♂ (NCA 2002/524); Same locality, leg. C. Haddad, 25.X.2003 (*Acacia erioloba* leaf litter), 1♂ (NCA 2002/513); Willem Pretorius Nature Reserve, 28°16.660'S, 27°12.207'E, leg. R. Fourie & A. Grobler, 30.IX–28.X.2009 (pitfall traps, near water level), 1♂ (NCA 2009/3526). *Gauteng Province*:Alice Glockner Nature Reserve, Wonderboom, Farm Rietspruit, 26°44.389'S, 28°22.661'E, leg. R. Koko, 2.VIII.2005 (pitfall traps), 1♀ (NCA 2008/2783); Johannesburg, Florida [26°30'S, 27°54'E], leg. R. Tucker, XII.1918, 1♀ (SAM B4507); Krugersdorp, Farm Hekpoort, 25°56'52.9''S, 27°37'46.0''E, 1447m a.s.l., leg. H. Roux, 2.XII.2003 (baited pitfall, open woodland), 1♀ (NCA 2008/4280); Marievale Bird Sanctuary, 26°20'S, 28°32'E, leg. V.D. & B. Roth, 8.XII.1990, 1♀ (CAS, CASENT 9033179). *KwaZulu-Natal Province*: Empangeni, 28°45'S, 31°54'E, leg. P. Reavell, 21.XI.1978 (on wall of plastic pool), 1♀ (NMSA); Hluhluwe-Imfolozi Park, Hilltop Research Station, 28°04.680'S, 32°02.472'E, leg. C. Haddad, 20.IV.2006 (leaf litter, Afromontane forest), 1imm. 1♂ 1♀ (NCA 2006/808); iSimangaliso [Greater St Lucia] Wetlands Park, Hell's Gate, 28°02.3'S, 32°26.0'E, leg. J. Esterhuizen, 26.I.2004 (tsetse fly traps), 1♂ (NCA 2004/776); Mfongozi, 27°17'S, 32°09'E, leg. W.C. Jones, I.1918, 1♂ (SAM B4140); Ndumo Game Reserve, Dipini Hide, 26°51.678'S, 32°15.514'E, leg. C. Haddad, 6.VII.2002 (on ground), 1♂ (NCA 2002/375); Same locality, E shore of Shokwe Pan, 26°52.516'S, 32°12.407'E, leg. C. Haddad, 22.I.2006 (grass at base of fever trees), 1♂ 2♀ (NCA 2006/721); Pietermaritzburg, 29°37'S, 30°23'E, leg. R.F. Lawrence, XI.1943, 2♀ (NMSA 3921); Same locality, leg. R.F. Lawrence, XII.1936, 1♂ (NMSA 1341); Umgeni River Valley [29°28'S, 30°14'E], leg. R.F. Lawrence, XII.1959, 1♀ (NMSA 7425); Zululand, 28°18'S, 32°21'E, leg. J. Pryke, 1.II.2010 (pitfall traps, indigenous forest), 1♀ (NCA 2011/910). *Limpopo Province*: Acornhoek [24°36'S, 31°05'E], leg. R. Tucker, XI.1918, 2imm. 4♂ 8♀ (SAM B4385); Bekendevlei, between Settlers and Tuinplaas (Springbokvlakte), leg. M. van Jaarsveld, 17.XII.2002 (pitfall traps, grass), 3♂ (NCA 2003/1337); Klein Kariba, near Warmbaths [Bela-Bela], 24°50'S, 28°20'E, 1140m a.s.l., leg. C.E. Griswold, 24–28.XI.1996 (lush bushveld), 1♀ (CAS, CASENT 9033184); Makalali Private Game Reserve, 24°09'S, 30°41'E, leg. C. Whitmore, XI.1999, 1♀ (NCA 2007/1150); Messina [Musina, 22°20'S, 30°02'E], leg. R. Tucker, XI.1918, 1♀ (SAM B4472); Roedtan, between Settlers and Tuinplaas (Springbokvlake), leg. M. van Jaarsveld, 6.II.2002 (pitfall traps, grass), 1♀ (NCA 2003/505); Settlers, 24°59'S, 28°33'E, leg. H. van der Merwe, 5.XII.1979, 1♀ (NCA 2010/246); Soutpansberg Mountains, Lajuma Mountain Retreat, Woodland 2a, 23°02.534'S, 29°26.848'E, leg. S. Foord, 25.I.2008 (pitfall traps), 1♀ (NCA 2008/1897); Same locality, leg. S. Foord, 3.XI.2004 (sifting litter, woodland), 1♂ (NCA 2010/2626). *Mpumulanga Province*: Bethal, 26°26'S, 29°27'E, leg. Dr. Broodryk, I.1986 (maize field), 1♂ (NCA 86/60); Delmas, Farm Rietvallei, 26.087°S, 28.573°E, leg. M. van Jaarsveld, 23.II.2005 (pitfall traps, unsprayed maize), 1♀ (NCA 2007/1303); Delmas, Farm Welgevonden, 26°14.640'S, 28°42.378'E, leg. D. Jacobs, 9–26.XII.2007, 1♂ (NCA 2008/3900); Komatipoort [25°31'S, 31°49'E], leg. R. Tucker, XI.1918, 1♀ (SAM B4342); Kruger National Park, 6km S of Skukuza, 25°00'S, 31°36'E, 1200ft, leg. C. Griswold, 17.XII.1984 (in shady ravine), 1♀ (NMSA 20278). *Northern Cape Province*: Kathu district, Pniel Farm, 28°35.420'S, 24°31.967'E, leg. R. Lyle, 20.IX–31.X.2005 (pitfall traps, dry savanna), 1imm. 1♂ (NCA 2006/1097); Prieska district, Green Valley Nuts, 29°35'S, 22°56'E, leg. C. Haddad, 18.XII.2002 (under cut ground cover vegetation, pistachio orchards), 1♂ 2♀ (NCA 2002/487); Schmidtsdrift district, Geelkoppies Farm, 28°43'S, 23°52'E, leg. C. Haddad, V.2002 (kukuyu grass along reservoir), 1♂ (NCA 2002/489). *North West Province*: Brits, 25°39'S, 27°45'E, leg. R. Watmough, 1984–1985 (cotton field), 2♀ (NCA 87/8); Vryburg district, Weltevrede Farm, 27°24.976'S, 24°29.906'E, leg. R. Lyle, R. Fourie, D. du Plessis & J. Adendorff, 9–12.I.2008 (leaf litter, Winkler traps), 1♀ (NCA 2009/3675); Same locality, 27°26.258'S, 24°29.873'E, leg. R. Lyle, R. Fourie, D. du Plessis & J. Adendorff, 10.I.2008 (leaf litter, dry river bed), 1♀ (NCA 2009/3677). *Western Cape Province*: Fisherhaven, near Hermanus, 34°21.430'S, 19°07.557'E, leg. C. Haddad, 12.I.2008 (sifting leaf litter), 1♀ (NCA 2008/462); Same locality, leg. C. Haddad, 26.XII.2000 (under rocks along Bot River Lagoon), 1♀ (NCA 2002/503); Malmesbury, Rondeberg, 33°24'S, 18°16'E, leg. G. Visagie, 24.X.1987 (under plants), 1♀ (NMBA 2143); Montagu Baths, 33°47'S, 20°07'E, leg. W.F. Purcell, X.1902, 1♂ 2♀ (SAM 12676); Swartberg Nature Reserve, Gamkaskloof, Die Hel, 33°21'S, 21°41'E, leg. Z. van der Walt, 15.II.2001 (on soil), 1♂ 3♀ (NCA 2002/202); Same data, 1♀ (NCA 2005/2029). SWAZILAND: Hlatikulu [26°57'S, 31°18'E], I.1939, 1♀ (NMSA 2574); Mbabane [26°19'S, 31°08'E], leg. R.F. Lawrence, XI.1964, 1imm. 2♂ 4♀ (NMSA 9441). TANZANIA: Mkomazi Game Reserve, behind Ibaya Camp, 04°00'S, 38°00'E, leg. S. van Noort, 1.XII.1993 (leaf litter near stream), 1♀ (SAM C5360); Same locality, Ibaya Camp, 04°00'S, 38°00'E, leg. S. van Noort, 6.XII.1993 (in *Ficus* litter), 1♂ (SAM C5359); Same locality, leg. A. Russell- Smith, 24.XI.1994 (litter of dry *Spirostachys* forest), 3♂ 8♀ (MRAC 211326); Uzungwa Mountains, Mwanihana Forest, Sanje River [07°50'S, 36°50'E], 300m a.s.l., loc. 14, leg. M. Stoltze & N. Scharff, 25.VIII.1982, 1♀ (ZMUC); 1km N of Matema, forest at foot of Livingstone Mountains, 09°30'S, 34°03'E, leg. R. Jocqué, 24.XI.1991 (sieved litter), 2♀ (MRAC 1735592); Same locality, leg. R. Jocqué, 5.XI.1991 (litter, by hand), 1♀ (MRAC 173449). UGANDA: Entebbe, Entebbe Botanical Gardens [00°03'N, 32°28'E], leg. A. Russell-Smith, 17.V.1991 (in long grass), 1♀ (PCRS); Mpanga Forest Reserve, near Mpigi [00°12'N, 32°17'E], leg. A. Russell-Smith, 28.VI.1998 (in litter), 1♀ (PCRS). ZIMBABWE: 42km S of Karoi, 1729B2, leg. Falcon College and NHMZ staff, 16.XII.1984, 1♀ (NMZ 3977); Bulawayo, Hillside, 20°10'S, 28°33'E, leg. M. FitzPatrick, II.1999, 1♀ (NMZ 15401); Same locality, leg. M. FitzPatrick, III.1999, 1♀ (NMZ 15405); Cheware River, 2km SW of Kasawe spring, 1629B1, leg. Falcon College and NHMZ staff, 8.XII.1984, 1♀ (NMZ 3850); Detema stream, 1km NE of Tobwe School, 1827B1, leg. I.M. Sango, 31.VIII.1985, 1♂ (NMZ 3741); S of Bulawayo, R.E.P. School, Matopos, 2028B3, leg. S. Higgins, 1.XI.1979, 1♀ (NMZ 536); Victoria Falls, 17°56'S, 25°50'E, leg. W.J. Pulawski, 1–8.II.1995, 4♀ (CAS, CASENT 9033117).

##### Diagnosis.

**Figures 80–83.**
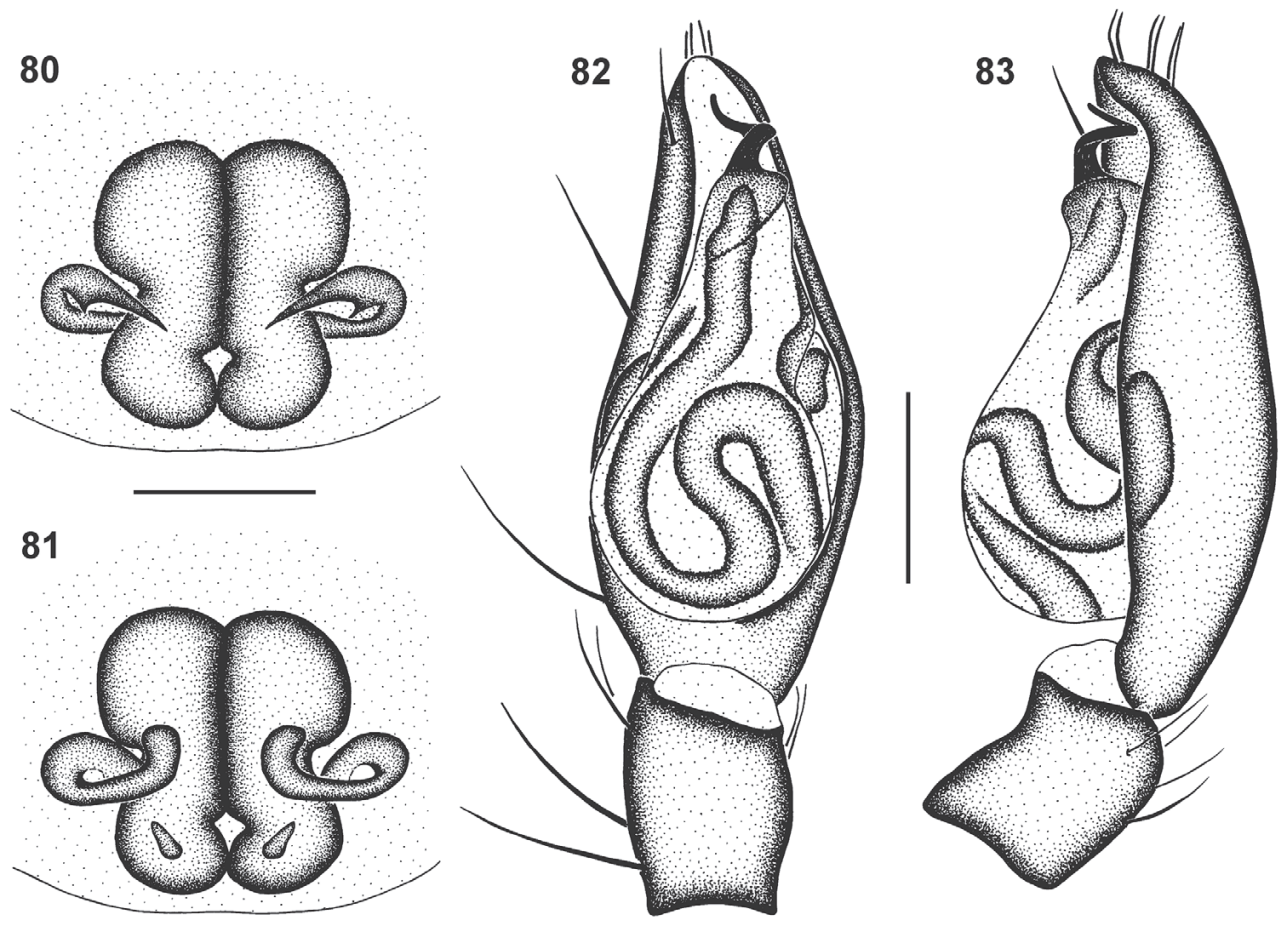
Genitalic morphology of *Cambalida fulvipes* (Simon, 1896): **80** female epigyne, ventral view **81** same, dorsal view **82** male palp, ventral view **83** same, retrolateral view. Scale bars = 0.25mm.

Females of this species can be recognised by the broad curved epigynal ridges and the nearly round ST II of the female epigyne (Fig. 80). Males have a curved basal section of the embolus and a distal section that is gently curved towards the tip of the embolus (Figs 54, 82).

##### Remarks.

The larger of the two females in the type series is designated here as the lectotype.

##### Female

**(Hopefield, NCA 2002/502).** Measurements: CL 2.45, CW 1.84, AL 3.70, AW 2.30, TL 5.95 (5.95–6.90), FL 0.22, SL 1.16, SW 1.08, AME–AME 0.07, AME–ALE 0.02, ALE–ALE 0.34, PME–PME 0.14, PME–PLE 0.07, PLE–PLE 0.54, PERW 0.77, MOQAW 0.32, MOQPW 0.44, MOQL 0.43.

Length of leg segments (sequence from femur to tarsus, and total): I 1.70 + 0.75 + 1.36 + 1.28 + 0.88 = 5.97; II 1.55 + 0.63 + 1.13 + 1.11 + 0.75 = 5.17; III 1.40 + 0.68 + 1.04 + 1.23 + 0.65 = 5.00; IV 2.09 + 0.85 + 1.85 + 2.12 + 0.85 = 7.76.

Carapace dark orange-brown with black mottling, clypeus slightly paler, eye region darker; black striae radiating from fovea towards palps and leg coxae; surface finely granulate, sparsely covered in white plumose setae. All eyes with black rings; AER procurved, ALE much larger than AME; AME separated by distance approximately ^3^∕_5_ their diameter, AME separated from ALE by ^1^∕_6_ AME diameter; clypeus height slightly less than 1½ AME diameter; PER procurved, PLE slightly larger than PME; PME separated by distance slightly less than their diameter, PME separated from PLE by distance slightly more than ½ PME diameter; CW:PERW = 2.39:1. Chelicerae deep orange-brown with black mottling on anterior surface, pale orange-brown proximally and along prolateral distal margin; three teeth on promargin, median tooth largest, proximal and distal teeth smaller and subequal, distal tooth situated closest to median tooth; two slightly separated subequal teeth on retromargin; endites mottled dark brown, fading to yellow and cream prolaterally; labium mottled dark brown, cream distally; sternum deep orange-brown with dark brown mottling, except at setal bases, giving speckled appearance. Legs finely granulate; femora I brown with black mottling, except along dorsal midline, yellow dorsally at distal end; femora II and III yellow with broad black mottled ring at ¾ their length; femora IV yellow, with dark brown ring with black mottling from ½ their length, yellow at distal end; patellae I–IV yellow with faint black mottling, dark around patellar indentation; tibiae, metatarsi and tarsi I–III yellow with faint lateral mottling; tibiae IV yellow-orange with broad incomplete mott- led black ring medially, mottling absent dorsally, proximally and distally; metatarsi IV yellow with faint black lateral mottling; tarsi IV yellow. Leg spination: femora: I pl 1 do 3, II do 3, III pl 2 do 3 rl 1, IV pl 1 do 3 rl 1; patellae with do 1 long distal seta; tibiae: I plv 1 rlv 1, II rlv 1-2, III pl 2 rl 2 plv 2 vt 2, IV pl 2 rl 2 plv 2 vt 2; metatarsi: I plv 2 rlv 2, II plv 2 rlv 2, III pl 2 rl 2 plv 2 rlv 2 vt 3, IV pl 3 rl 3 plv 2 rlv 2 vt 3. Palpal spination: femora do 2, patellae pl 1 spine and do 2 short setae, one proximally and one distally, tibiae pl 1 do 1 plv 1, tarsi pl 1 plv 3 rlv 1. Abdomen mottled dark grey, with cream spots around sigilla, fine cream chevrons in posterior ⅔ of abdomen and small white spot above spinnerets; dorsal scutum mottled dark brown, extending ^1^∕_5_ abdomen length; venter mottled pale grey, darker towards spinnerets, epigastric sclerite brown and inframamillary sclerite yellow-brown. Epigyne with broad curved epigynal ridges with lateral copulatory openings (Fig. 80); copulatory ducts initially directed laterally, looping sharply towards posterior then transversely towards midline, bending at nearly a right angle before entering ST II posteromedially; ST II somewhat round, joined broadly to large kidney-shaped posterior ST I (Fig. 81).

##### Male

**(Bain’s Vlei, TMSA 24131).** Measurements: CL 2.50, CW 1.83, AL 2.75, AW 1.55, TL 5.10 (5.07–5.45), FL 0.19, SL 1.10, SW 1.01, AME–AME 0.07, AME–ALE 0.03, ALE–ALE 0.34, PME–PME 0.13, PME–PLE 0.06, PLE–PLE 0.52, PERW 0.73, MOQAW 0.33, MOQPW 0.42, MOQL 0.41.

Length of leg segments (sequence from femur to tarsus, and total): I 1.73 + 0.73 + 1.48 + 1.43 + 1.03 = 6.40; II 1.62 + 0.63 + 1.23 + 1.26 + 0.91 = 5.65; III 1.49 + 0.65 + 1.10 + 1.35 + 0.73 = 5.32; IV 2.19 + 0.81 + 1.94 + 2.20 + 0.95 = 8.09.

Carapace dark brown, nearly black, clypeus black medially and paler brown laterally, eye region black; black striae radiating from fovea towards palps and leg coxae; surface finely granulate, densely covered in white plumose setae. All eyes with black rings; AER procurved, ALE larger than AME; AME separated by distance slightly more than ^3^∕_5_ their diameter, AME separated from ALE by distance equal to ¼ AME diameter; clypeus height equal to 1^4^∕_5_ AME diameter; PER procurved, PLE slightly larger than PME; PME separated by distance slightly more than ⅞ their diameter, PME separated from PLE by distance slightly more than ^2^∕_5_ PME diameter; CW:PERW = 2.51:1. Chelicerae dark brown with black mottling on anterior surface, yellow along prolateral distal margin; three teeth on promargin, median tooth largest, proximal and distal teeth smaller and subequal, distal tooth situated closest to median tooth; two slightly separated subequal teeth on retromargin, closer to fang base than promarginal teeth; endites dark brown, fading to yellow and cream prolaterally; labium dark brown, cream distally; sternum deep red-brown with black mottling, except at setal bases, giving speckled appearance. Legs finely granulate; femora I dark brown, bright yellow-orange dorsally and at distal end laterally; femora II bright orange with proximal and lateral black mottling; femora III orange with black mottling in distal ⅓; femora IV dark orange with black lateral mottling and broad black band in distal ½; patellae I–III bright yellow and IV bright orange, all with black lateral mottling, darker around patellar indentation; tibiae, metatarsi and tarsi I and II yellow and III orange with faint black lateral mottling; tibiae IV reddish-orange with dense black mottling, yellow at distal end; metatarsi IV bright red-orange with black mottling, except at proximal end; tarsi IV yellow-orange. Leg spination: femora: I pl 1 do 3, II pl 1 do 3, III pl 2 do 3 rl 1, IV pl 2 do 3 rl 1; patellae with do 1 long distal seta; tibiae: I plv 1 rlv 1, II rlv 2, III pl 2 rl 2 plv 1-2 rlv 0-1 vt 2, IV pl 2 rl 2 plv 2 rlv 1 vt 2; metatarsi: I plv 2 rlv 2, II plv 2 rlv 2, III pl 2 rl 2 plv 2 rlv 2 vt 3, IV pl 3 rl 3 plv 2 rlv 2 vt 3. Palpal spination: femora pl 1 do 2, patellae pl 1 spine and do 2 short setae, one proximally and one distally, tibiae pl 1 plv 1, tarsi pl 2 plv 2. Abdomen with deep wine-red dorsal scutum with dense black mottling, covering entire dorsum; small white spot of dense plumose setae just above spinnerets; venter mottled dark grey, epigastric sclerite, post-epigastric sclerites and ventral sclerite deep red-brown, inframamillary sclerite yellow-brown. Palps dark brown with dense black mottling; embolus short and broad, basal coil slightly curved, distal section nearly transverse in ventral view, curved towards tip (Figs 54, 82, 83).

##### Distribution.

Widely distributed throughout sub-Saharan Africa (Fig. 95).

##### Biology.

This is the *Cambalida* species that occupies the greatest range of habitats, from tropical and temperate forests, to savannas, grasslands, karoo and fynbos. Although generally scarce in agroecosystems, this species has been caught in the ground cover layer of pistachio nuts in the Northern Cape Province of South Africa (Haddad and Dippenaar-Schoeman 2006), maize fields in Kenya and South Africa, and rice paddies in West Africa.

Feeding in *Cambalida fulvipes* follows a similar pattern to that observed for other corinnids such as *Graptartia* (Haddad 2004). Prey is grasped using the first two pairs of legs, which form a basket in which the prey is subdued. Following the bite, prey may die within 1 minute (e.g. vinegar flies *Drosophila melanogaster* Meigen), after which feeding commences. Once complete, only a small ball of macerated prey remains is left.

#### Cambalida griswoldi sp. n.

urn:lsid:zoobank.org:act:DC379B57-CF65-4DC1-8D42-901E4A6DD3E5

http://species-id.net/wiki/Cambalida_griswoldi

Figures 55 , 84–86

##### Type material.

**Holotype female.** MADAGASCAR:*Antsiranana*: Réserve Spéciale d’Ambre, 3.5km 235°SW Sakaramy, 12°28'08"S, 49°14'32"E, 325m a.s.l., leg. Fisher-Griswold Arthropod Team, 26–31.I.2001 (sifted litter, tropical dry forest) (CAS, CASENT 9006738).

##### Paratypes.

MADAGASCAR:*Antsiranana*: Forêt d’Orangea, 3.6km 128° SE Remena, 12°15'32"S, 49°22'29"E, 90m a.s.l., leg. Fisher, Griswold *et al*., 22–28.II.2001 (pitfall trap, littoral rainforest), 2♀ (CAS, CASENT 9007088); Same locality as holotype, leg. L.J. Boutin, 26–31.I.2001, 1♀ (CAS, CASENT 9000791). *Toliara*: 18km NNW Betroka, 23°09'48"S, 45°58'07"E, 825m a.s.l., leg. M. Ivie & A. Pollock, 9–14.XII.1994 (flight intercept traps), 1♂ (CAS, CASENT 9033123).

##### Other material examined.

None.

##### Diagnosis.

**Figures 84–88.**
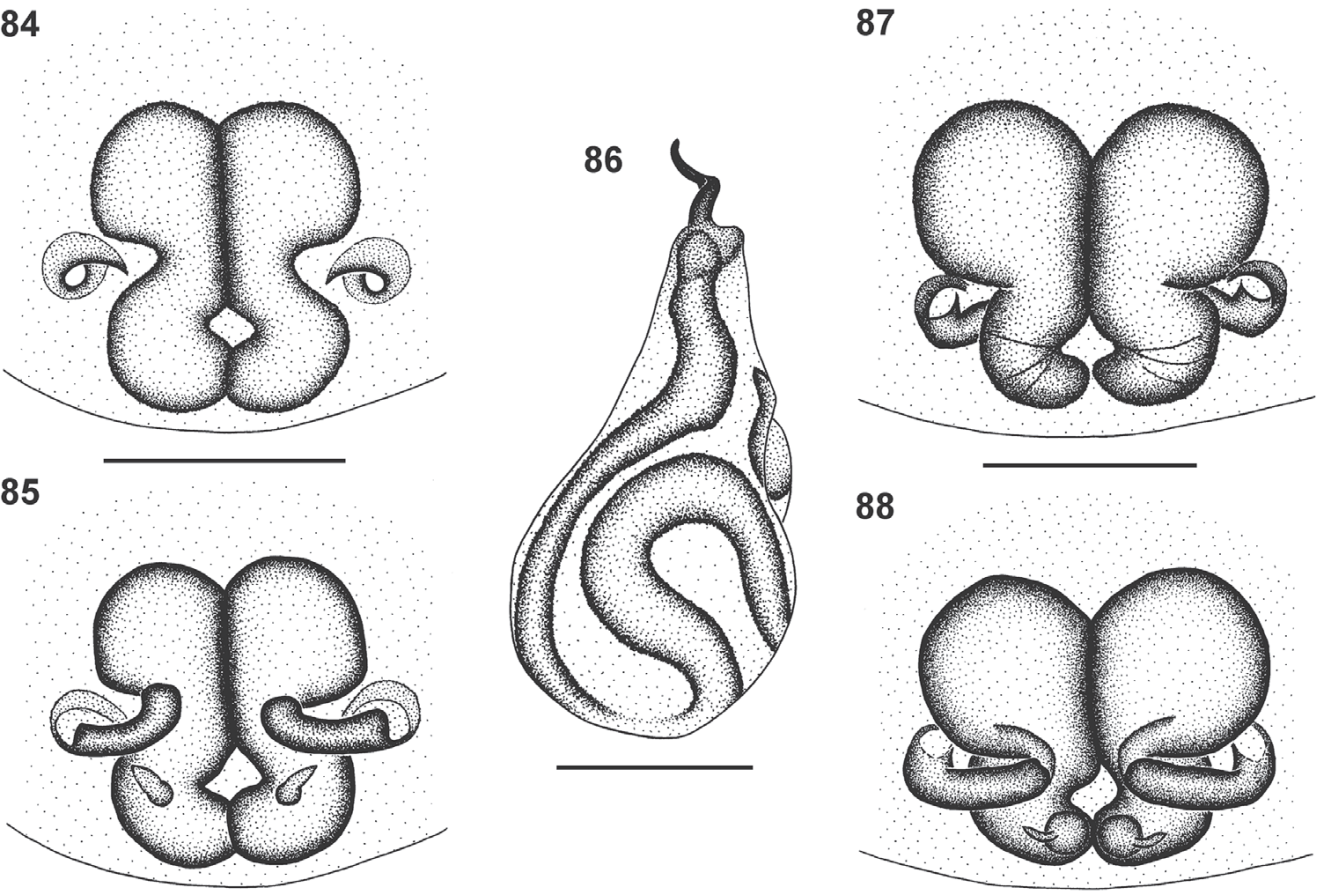
Genitalic morphology of *Cambalida griswoldi* sp. n. **(84–86)** and *Cambalida lineata* sp. n. **(87, 88)**: **84, 87** female epigyne, ventral view **85, 88** same, dorsal view **86** male palpal tegulum, ventral view. Scale bars = 0.25mm.

Females have similar spermathecal proportions to the continental *Cambalida fulvipes* but can be recognised by the narrower, distinctly coiled epigynal ridges (Fig. 84), which are broad and curved in *Cambalida fulvipes* (Fig. 80). The distal section of the male embolus is short and slightly curved towards the tip (Fig. 86).

##### Etymology.

A patronym in honour of Charles Griswold, one of the collectors of the types, in recognition of his vast and significant collections of spiders on Madagascar.

##### Remark.

The palp of the paratype male is greatly expanded and only the tegulum is illustrated here (Fig. 86). The cymbium has the distal setae characteristic of the genus, confirming the placement of this species and the occurrence of *Cambalida* on Madagascar.

##### Female

**(holotype, Réserve Spéciale d’Ambre, CASENT 9006738).** Measurements: CL 2.10, CW 1.52, AL 2.53, AW 1.78, TL 4.70 (4.65–4.75), FL 0.13, SL 0.97, SW 0.89, AME–AME 0.05, AME–ALE 0.02, ALE–ALE 0.26, PME–PME 0.11, PME–PLE 0.06, PLE–PLE 0.43, PERW 0.62, MOQAW 0.23, MOQPW 0.37, MOQL 0.36.

Length of leg segments (sequence from femur to tarsus, and total): I 1.28 + 0.57 + 1.03 + 0.98 + 0.64 = 4.50; II 1.17 + 0.55 + 0.84 + 0.88 + 0.56 = 4.00; III 1.05 + 0.54 + 0.70 + 0.95 + 0.44 = 3.68; IV 1.48 + 0.61 + 1.28 + 1.57 + 0.58 = 5.52.

Carapace deep orange-brown, clypeus deep yellow-brown, eye region slightly darker; faint black striae radiating from fovea towards palps and coxae and faint black mottling on slopes; surface finely granulate, sparsely covered in white plumose setae. AER procurved, eyes subequal in diameter; AME separated by distance ½ their diameter, AME separated from ALE by distance equal to ¼ AME diameter; clypeus height equal to 1⅔ AME diameter; PER procurved, PLE slightly larger than PME; PME separated by distance slightly more than ^4^∕_5_ their diameter, PME separated from PLE by distance slightly less than ½ PME diameter; CW:PERW = 2.45:1. Chelicerae deep yellow-brown with black mottling on anterior surface; three teeth on promargin, median tooth largest, proximal and distal teeth small, subequal in size, median and distal teeth adjacent; two teeth on retromargin, distal tooth slightly larger than proximal tooth, closer to fang base than promarginal teeth; endites yellow with black mottling, cream prolaterally; labium orange-brown with faint black mottling, cream distally; sternum orange with dense brown mottling, except at setal bases. Legs finely granulate; femora I–IV yellow-brown with black mottling laterally and ventrally, absent dorsally and at distal end ventrally; patellae I–IV yellow-brown with black mottling laterally and ventrally, absent dorsally, with faint median dorsal black line; tibiae I yellow with black mottling in distal half; tibiae II and III yellow-brown and IV orange-brown, all with black mottling, faint dorsally, with fine median dorsal black line; metatarsi I–III yellow with black mottling, absent proximally; metatarsi IV yellow-brown with black mottling, faint dorsally, absent proximally and distally; tarsi I–IV yellow. Leg spination: femora: I pl 1 do 3, II do 1, III pl 1 do 2 rl 1, IV pl 1 do 3 rl 1; patellae with do 1 long distal seta; tibiae: I plv 3 rlv 2, II rlv 2, III pl 2 rl 2 plv 2 rlv 1, IV pl 2 rl 2 plv 2 rlv 1 vt 2; metatarsi: I plv 2 rlv 2, II plv 2 rlv 2, III pl 2 rl 2 plv 2 rlv 2 vt 3, IV pl 3 rl 3 plv 2 rlv 2 vt 3. Palpal spination: femora do 2, patellae pl 1, tibiae pl 1 plv 1, tarsi pl 1 plv 3. Abdomen mottled dark grey dorsally with fine cream chevrons posteriorly; dorsum with red-brown scutum extending ⅔ abdomen length, with black mottling darker around margins of scutum, forming ring-shaped dorsal marking; venter mottled pale grey, darker towards spinnerets, with paired rectangular black markings between paired sclerite rows, midway between epigastric furrow and spinnerets; epigastric sclerite red-brown and inframamillary sclerite yellow-brown. Epigyne with small lateral copulatory openings situated within 6-shaped epigynal ridges (Fig. 84); copulatory ducts initially directed transversely medially, bending at nearly right angle, entering ST II posterolaterally; ST II round, with nearly right-angled posterolateral margin, joined broadly to kidney-shaped posterior ST I (Fig. 85).

##### Male

**(paratype, Betroka, CASENT 9033123).** Measurements: CL 2.23, CW 1.72, AL 2.70, AW 1.61, TL 4.83, FL 0.15, SL 1.03, SW 0.94, AME–AME 0.06, AME–ALE 0.02, ALE–ALE 0.30, PME–PME 0.13, PME–PLE 0.06, PLE–PLE 0.48, PERW 0.71, MOQAW 0.29, MOQPW 0.41, MOQL 0.44.

Length of leg segments (sequence from femur to tarsus, and total): I 1.53 + 0.63 + 1.30 + 1.24 + 0.95 = 5.65; II 1.38 + 0.60 + 1.05 + 1.08 + 0.81 = 4.92; III 1.23 + 0.57 + 0.90 + 1.11 + 0.65 = 4.46; IV 1.89 + 0.74 + 1.69 + 1.90 + 0.83 = 7.05.

Carapace dark brown, eye region darker; faint black striae radiating from fovea towards palps and leg coxae; surface finely granulate, densely covered in white plumose setae. Rings around eyes faded to orange-brown; AER procurved, ALE larger than AME; AME separated by distance equal to ½ their diameter, AME separated from ALE by ^1^∕_5_ AME diameter; clypeus height slightly less than 1½ AME diameter; PER procurved, PLE slightly larger than PME; PME separated by distance slightly less than ¾ their diameter, PME separated from PLE by distance slightly less than ⅓ PME diameter; CW:PERW = 2.42:1. Chelicerae brown with faint black mottling on anterior surface, yellow prolaterally in distal half; three teeth on promargin, median tooth largest, distal tooth smallest, median and distal teeth closest; two slightly separated teeth on retromargin, distal tooth slightly smaller than proximal tooth, closer to fang base than promarginal teeth; endites pale brown with dark brown mottling, cream prolaterally; labium pale brown with dark brown mottling, cream distally; sternum red-brown with dark brown mottling. Legs finely granulate; femora I–IV dark brown, slightly paler dorsally, yellow dorsally at distal end; patellae I–IV yellow with black mottling laterally and ventrally distally, darker on posterior legs; tibiae I and II yellow with black mottling; tibiae III and IV orange-brown with dense black mottling, paler along dorsal midline; metatarsi I and II yellow-brown, with sparse dark brown mottling laterally; metatarsi III and IV dark brown, yellow at proximal and distal ends; tarsi I–IV yellow, tarsus IV with black mottling medially. Leg spination: femora: I pl 1 do 3, II do 3, III pl 1 do 3 rl 1, IV pl 1 do 3 rl 1; patellae with do 1 long distal seta; tibiae: I and II spineless, III pl 2 rl 2 plv 2, IV pl 2 rl 2 plv 2 vt 2; metatarsi: I plv 2 rlv 1, II plv 1 rlv 1, III pl 2 rl 1 plv 1 vt 3, IV pl 3 rl 3 plv 2 vt 3. Palpal spination: femora pl 1 do 2, patellae pl 1, tibiae pl 1 plv 1, tarsi pl 1 plv 3. Abdomen with dark red-brown, nearly black scutum covering entire dorsum; venter mottled pale grey between sclerites; epigastric sclerite, post-epigastic sclerites and ventral sclerite deep red-brown with black mottling, inframamillary sclerite orange-brown. Palps orange-brown with dense black mottling; embolus short, basal coil slightly curved, distal section gently curved towards tip (Fig. 86).

##### Distribution.

Known from isolated localities in northern and southern Madagascar (Fig. 95).

##### Biology.

A ground-dwelling species collected in contrasting forest types.

#### Cambalida lineata sp. n.

urn:lsid:zoobank.org:act:59D31562-99C6-4A9A-B5A3-500793263E3A

http://species-id.net/wiki/Cambalida_lineata

Figures 7 , 87, 88

##### Type material.

**Holotype female.** MADAGASCAR: *Toamasina*: Parc National Masoala, Ambohitsitondroina Mountain, Ambanizana, 15°34'09.9"S, 50°00'12.3"E, 600–650m a.s.l., leg. D. Andriamalala, D. Silva *et al*., 1–2.III.2003 (rainforest, general collecting, night) (CAS, CASENT 6015409).

##### Paratypes.

MADAGASCAR: *Toamasina*: Presqu’ile de Masoala, 5.3km SSE Ambanizana, Andranobe, 15°40'S, 49°58'E, 425m a.s.l., leg. B.L. Fisher, 21.XI.1993 (sifted litter, leaf mould, rotten wood, rainforest), 1♀ (CAS, CASENT 9033140); Same locality as holotype, 750–800m a.s.l., leg. D. Andriamalala, D. Silva *et al*., 1.III.2003 (rainforest, sweeping), 1♀ (CAS, CASENT 6015423). *Toliara*: Réserve Spéciale d’Ambohijanahary, Forêt d’Ankazotsihitafototra, 35.2km 312° NW Ambaravaranala, 18°16'00"S, 45°24'24"E, 1050m a.s.l., leg. Fisher, Griswold *et al*., 13–17.I.2003 (general collecting, day spiders), 1♀ (CAS, CASENT 9012850).

##### Other material examined.

None.

##### Diagnosis.

The species is unique amongst *Cambalida* in the cream median stripe on the abdomen, which extends to the carapace in the holotype (Fig. 7) and one of the paratypes, but is very indistinct in two of the paratypes. The ST II are very large and round, and nearly twice as broad as the ST I (Figs 87, 88).

##### Etymology.

From the Latin “linea”, referring to the pale dorsal stripe on the abdomen.

##### Female

**(holotype, Parc National Masoala, CASENT 6015409).** Measurements: CL 2.50, CW 1.74, AL 2.65, AW 1.65, TL 5.20 (5.05–5.60), FL 0.20, SL 1.19, SW 1.06, AME–AME 0.06, AME–ALE 0.02, ALE–ALE 0.31, PME–PME 0.11, PME–PLE 0.05, PLE–PLE 0.48, PERW 0.71, MOQAW 0.30, MOQPW 0.44, MOQL 0.41.

Length of leg segments (sequence from femur to tarsus, and total): I 1.96 + 0.79 + 1.74 + 1.66 + 1.05 = 7.20; II 1.90 + 0.74 + 1.55 + 1.55 + 0.85 = 6.59; III 1.75 + 0.71 + 1.32 + 1.69 + 0.80 = 6.27; IV 2.33 + 0.83 + 2.09 + 2.56 + 1.00 = 8.81.

Carapace orange-brown, clypeus slightly paler laterally, eye region slightly darker, with slightly paler yellow-orange line from fovea to posterior margin; black striae radia- ting from fovea towards palps and coxae and faint black mottling on slopes; surface finely wrinkled, sparsely covered in white plumose and short straight setae. All eyes with black rings; AER procurved, laterals larger than medians; AME separated by distance slightly less than ½ their diameter, AME separated from ALE by distance equal to ^1^∕_5_ AME diameter; clypeus height slightly larger than 1½ times AME diameter; PER procurved, PME and PLE equal in diameter; PME separated by distance slightly less than ⅔ their diameter, PME separated from PLE by distance slightly less than ⅓ PME diameter; CW:PERW = 2.45:1. Chelicerae deep orange-brown with black mottling on anterior surface, yellow along prolateral distal margin; bent setae on promargin pectinate; three teeth on promargin, median tooth largest, proximal tooth smallest, median tooth closer to distal tooth than to proximal tooth; two teeth on retromargin, distal tooth slightly larger than proximal tooth, closer to fang base than promarginal teeth; endites pale orange with faint black mottling, cream prolaterally; labium orange, paler distally; sternum bright orange with faint black mottling. Legs finely granulate; femora I–IV mottled brown, with paler dorsal lines proximally and distally and club-shaped retrolateral paler line; patellae I–IV brown, yellow-brown dorsally; tibiae I yellow, mott- led proximally and laterally at distal end; tibiae II–IV yellow dorsally and ventrally, mottled brown laterally, tibiae IV yellow at distal end; metatarsi and tarsi I–IV yellow with faint lateral brown mottling proximally. Leg spination: femora: I pl 1 do 2, II do 2, III pl 1 do 3, IV pl 1 do 3-4 rl 1; patellae with do 1 long distal seta; tibiae: I plv 3 rlv 2, II plv 2 rlv 2, III pl 2 do 1 rl 2 plv 2 rlv 2 vt 2, IV pl 2 do 1 rl 2 plv 2 rlv 1 vt 2; metatarsi: I plv 2 rlv 2, II plv 2 rlv 2, III pl 3 rl 3 plv 2 rlv 2 vt 3, IV pl 3 rl 3 plv 2 rlv 2 vt 3. Palpal spination: femora pl 1 do 2, patellae pl 1, tibiae pl 1 plv 1, tarsi pl 2 plv 2 rlv 1. Abdomen mottled dark grey dorsally, with narrow triangular median cream line, broadest anteriorly and extending past middle of abdomen, cream spots surrounding sigilla, and small white spot above spinnerets (Fig. 7); dorsum with yellow scutum extending ¼ abdomen length; sides of abdomen mottled dark grey, with cream line extending ⅔ the distance from epigastric furrow to spinnerets; venter mottled pale grey, darker towards spinnerets, epigastric sclerite and inframamillary sclerite yellow. Epigyne with small lateral copulatory openings situated within hemispherical epigynal ridges (Fig. 87); copulatory ducts initially directed posteriorly, bending at nearly right angle before running transversely towards midline, entering ST II posteromedially; ST II large and round, joined broadly to narrower kidney-shaped posterior ST I (Fig. 88).

##### Male.

Unknown.

##### Distribution.

Known from three isolated localities in northern and central Madagascar (Fig. 95).

##### Biology.

A ground-dwelling species collected in rainforest.

#### Cambalida loricifera

(Simon, 1885)

http://species-id.net/wiki/Cambalida_loricifera

**Figures 89–92.**
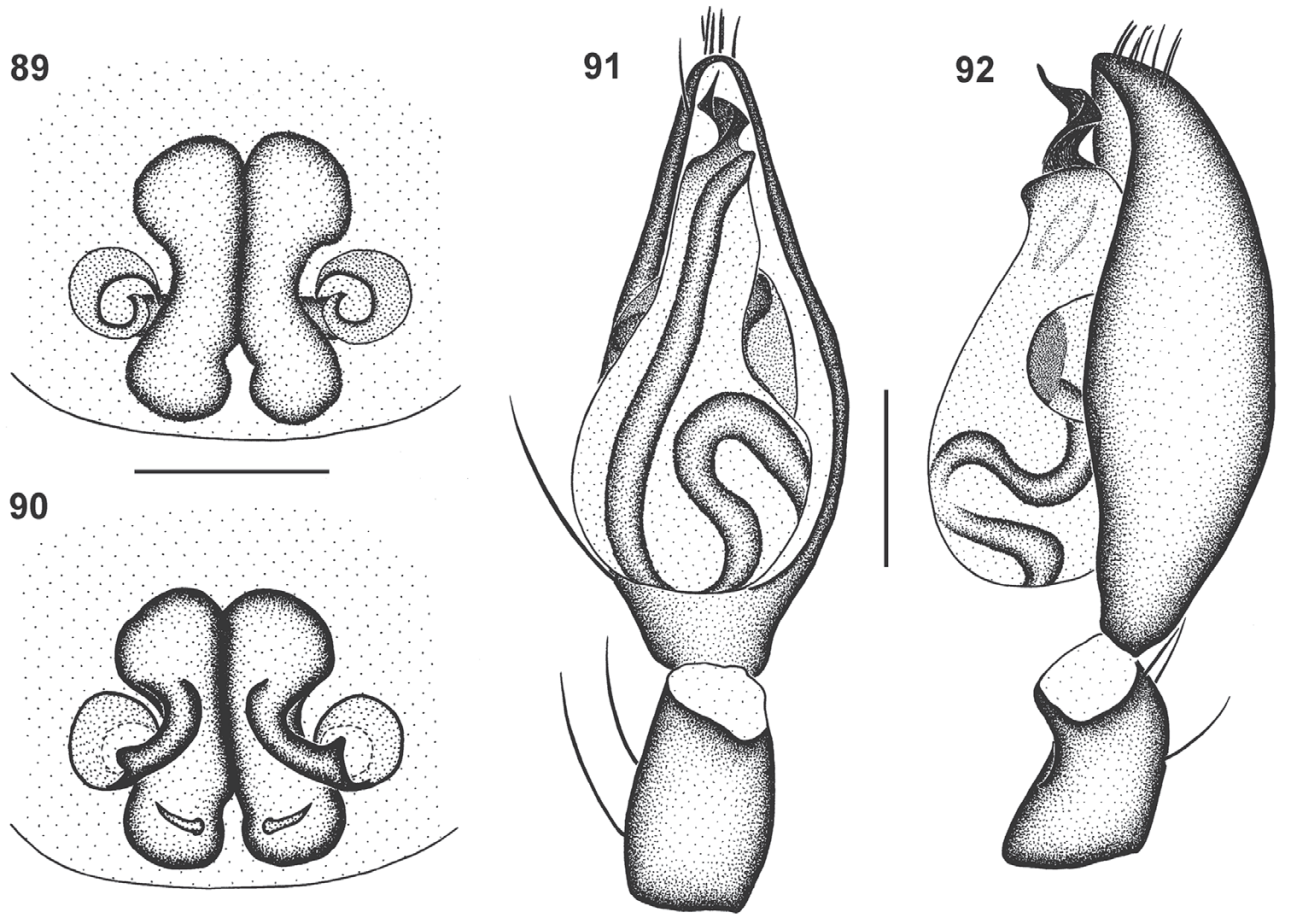
Genitalic morphology of *Cambalida loricifera* (Simon, 1885): **89** female epigyne, ventral view **90** same, dorsal view **91** male palp, ventral view **92** same, retrolateral view. Scale bars = 0.25mm.

Figures 43 , 56 , 89–92

Tylophora loricifera Simon, 1885: 379 comb. n.
Castaneira loricifera Simon, 1897: 167.

##### Type material.

**Holotype male.** SENEGAL: Dakar [14°45'N, 17°20'W], MNHN 8062 (examined).

##### Other material examined.

SENEGAL: Sonkorong [13°46'N, 15°33'W], near Kaymor, leg. A. Russell-Smith, 14.VI.1994 (leaf litter, 20 year old fallow), 4♂ (BMNH); Thyssé Region, Forêt Classé de Ngayene [13°43'N, 15°27'W], leg. A. Russell-Smith, 24.VII.1996 (leaf litter), 1♂ 3♀ (BMNH).

##### Diagnosis.

This species is recognised by the distinctly coiled epigynal ridges and the oblique curved entrance ducts of the females (Figs 89, 90). Males can be recognised by the broad, somewhat flattened embolus (Figs 43, 56).

##### Remarks.

The right leg I and right palp of the male holotype are missing. The redescription of the male is provided for the holotype, although more recently collected specimens are darker brown in colour.

##### Female

**(Ngayene, BMNH).** Measurements: CL 2.80, CW 2.05, AL 4.55, AW 2.25, SL 1.32, SW 1.21, TL 7.10 (6.45–7.10), AME–AME 0.06, AME–ALE 0.01, ALE–ALE 0.40, PME–PME 0.15, PME–PLE 0.06, PLE–PLE 0.61, PERW 0.87, MOQAW 0.39, MOQPW 0.52, MOQL 0.49.

Length of leg segments (sequence from femur to tarsus, and total): I 1.87 + 0.83 + 1.58 + 1.48 + 0.92 = 6.68; II 1.72 + 0.80 + 1.30 + 1.34 + 0.81 = 5.97; III 1.65 + 0.78 + 1.20 + 1.54 + 0.65 = 5.82; IV 2.31 + 0.95 + 2.02 + 2.48 + 0.90 = 8.66.

Carapace dark orange-brown, ocular region slightly darker, with dark brown mott- ling and black striae radiating from fovea towards palps and leg coxae; surface finely granulate, covered in white plumose setae, denser laterally. All eyes with black rings; AER procurved, AME and ALE subequal in size; AME separated by distance slightly less than ½ their diameter, AME separated from ALE by distance equal to ^1^∕_5_ AME diameter; clypeus height slightly larger than AME diameter; PER procurved, PME slightly larger than PLE; PME separated by distance equal to ^4^∕_5_ their diameter, PME separated from PLE by distance equal to ⅓ PME diameter; CW:PERW = 2.35:1. Chelicerae orange-brown, yellow prolaterally at distal end; three teeth on promargin, median tooth largest, distal tooth smallest, situated closest to median tooth; two teeth on retromargin, subequal in size, closer to fang base than promarginal teeth; endites orange, cream prolaterally; labium orange-brown, cream distally; sternum orange, with pale brown mottling; surface finely granulate, with scattered small, erect black setae; precoxal triangles indistinct, intercoxal sclerites present between coxae I and II, coxae II and III, coxae III and IV. Legs finely granulate; femora I–IV brown, yellow at distal end, with dark brown mottling laterally and darker band at ⅔ femora length; remaining segments of legs I–III yellow with dark brown lateral mottling, leg III slightly darker yellow than anterior legs; patellae IV yellow-brown with dark brown lateral mottling; tibiae IV brown, yellow at proximal and distal ends, with dark brown mottling laterally; metatarsi IV yellow brown dorsally, with brown mottling laterally and ventrally, slightly paler proximally and distally; tarsi IV yellow. Leg spination: femora: I pl 1 do 3, II pl 1 do 3, III pl 2 do 3 rl 1, IV pl 1 do 3 rl 1; patellae with single long distal do seta; tibiae: I plv 1 rlv 1, II rlv 2, III pl 2 do 1 rl 2 plv 1 rlv 1 vt 2, IV pl 2 do 1 rl 2 plv 2 rlv 1 vt 2; metatarsi: I plv 2 rlv 2, II plv 2 rlv 2, III pl 2 rl 2 plv 2 rlv 2 vt 3, IV pl 3 rl 3 plv 2 rlv 2 vt 3; all tibiae, metatarsi and tarsi with 4–10 do tr. Palpal spination: femora do 2, patellae pl 1 do 2, tibiae pl 2 do 2, tarsi pl 1 rl 1 plv 2 vt 2. Abdomen pale grey with scattered white median markings, with white spot above spinnerets; dorsum with orange dorsal scutum extending ¼ abdomen length; venter mottled pale grey, epigastric sclerite orange and inframamillary sclerite pale yellow-brown. Epigyne with small lateral copulatory openings situated prolaterally within broad comma-shaped epigynal ridges (Fig. 89); copulatory ducts initially directed transversely, curving obliquely towards anterior, entering ST II posterolaterally; ST II small and subtriangular with rounded angles, joined broadly to narrow kidney-shaped posterior ST I (Fig. 90).

##### Male

**(holotype, Dakar, MNHN 8062).** Measurements: CL 2.51, CW 1.86, AL 2.78, AW 1.50, TL 5.40 (4.60–5.40), FL 0.21, SL 1.16, SW 1.00, AME–AME 0.06, AME–ALE 0.01, ALE–ALE 0.34, PME–PME 0.14, PME–PLE 0.05, PLE–PLE 0.54, PERW 0.79, MOQAW 0.33, MOQPW 0.47, MOQL 0.44.

Length of leg segments (sequence from femur to tarsus, and total): I 1.64 + 0.68 + 1.34 + 1.25 + 0.85 = 5.70; II 1.46 + 0.62 + 1.10 + 1.11 + 0.70 = 4.99; III 1.40 + 0.68 + 0.96 + 1.26 + 0.61 = 4.91; IV 2.00 + 0.80 + 1.48 + 2.05 + 0.80 = 7.13.

Carapace orange-brown, including ocular region; surface finely granulate, cove- red in white plumose setae, denser laterally. All eyes with black rings; AER procurved, ALE slightly larger than AME; AME separated by distance slightly less than ½ their diameter, AME separated from ALE by approximately ^1^∕_5_ AME diameter; clypeus height equal to 1¼ AME diameter; PER procurved, PME slightly larger than PLE; PME separated by distance equal to ^4^∕_5_ their diameter, PME separated from PLE by distance equal to ⅓ PME diameter; CW:PERW = 2.35:1. Chelicerae orange-brown, yellow prolaterally at distal end; three teeth on promargin, median tooth largest, distal tooth smallest, situated closest to median tooth; two subequal teeth on retromargin, closer to fang base than promarginal teeth; endites orange, cream prolaterally; labium orange-brown, cream distally; sternum orange with pale brown mottling. Legs finely granulate; femora I–IV brown, yellow distally; patellae I–IV yellow, with faint brown mottling laterally; tibiae I and II yellow with brown mottling laterally; tibiae III and IV yellow-brown with brown lateral mottling, yellow distally; metatarsi I and II yellow, III and IV yellow-brown; tarsi I and II yellow, III and IV creamy-yellow. Leg spination: femora: I pl 1 do 3, II pl 1 do 3, III pl 2 do 3 rl 2, IV pl 2 do 3 rl 1; patellae with single long distal do seta; tibiae: I plv 1 rlv 1, II rlv 1, III pl 2 do 1 rl 2 plv 2 rlv 2 vt 2, IV pl 2 do 1 rl 2 plv 2 rlv 2 vt 2; metatarsi: I plv 2 rlv 2, II plv 2 rlv 2, III pl 2 rl 2–3 plv 2 rlv 2 vt 3, IV pl 3 rl 3 plv 2 rlv 2 vt 3. Palpal spination: femora pl 1 do 2, patellae do 1, tibiae pl 1 plv 1, tarsi plv 2. Abdomen with orange-brown dorsal scutum covering entire dorsum, yellow-brown posteriorly, with faint brown mottling; white spot comprising dense plumose setae above spinnerets; venter cream, epigastric sclerite, post-epigastric sclerites orange-brown, ventral sclerite and inframamillary sclerite orange. Palps yellow with brown mottling; embolus broadly coiled, somewhat flattened, with 1¼ coils (Figs 43, 91, 92).

##### Distribution.

Only known from three localities in Senegal, West Africa (Fig. 95).

##### Biology.

Collected from leaf litter in forests and fallow habitats.

#### Cambalida unica sp. n.

urn:lsid:zoobank.org:act:2F175096-C8FD-4936-B4FB-639E74C179DB

http://species-id.net/wiki/Cambalida_unica

Figures 93, 94

##### Type material.

**Holotype female, together with 1 paratype female.** CAMEROON: Chabal Mbabo, SW slope, 07°25'N, 12°49'E, 1250m a.s.l., leg. Bosmans & Van Stalle, 9.IV.1983 (grassland) (MRAC 162219).

##### Other material examined.

None.

##### Diagnosis.

**Figures 93–94.**
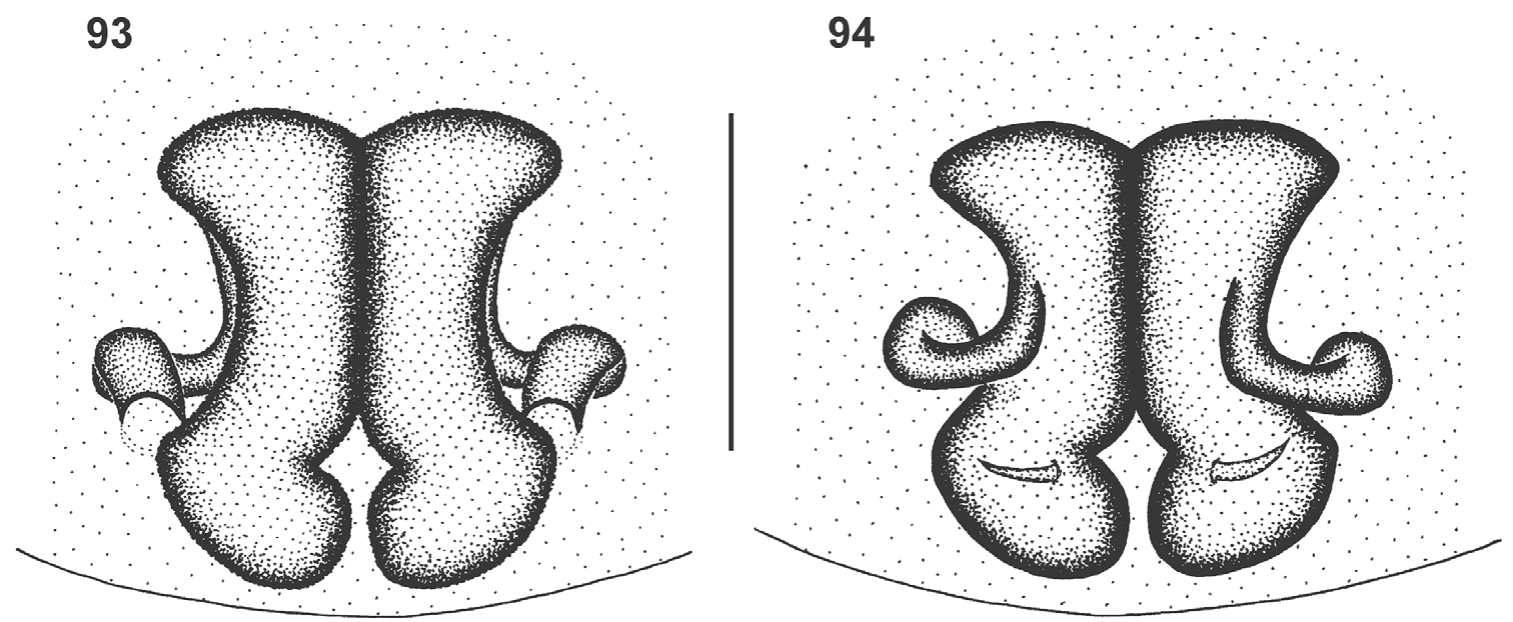
Genitalic morphology of *Cambalida unica* sp. n.: **93** female epigyne, ventral view **94**  same, dorsal view. Scale bar = 0.25mm.

Females of this species can be easily recognised by the sharply bent lateral margins of the ST II (Figs 93, 94).

##### Etymology.

The species name is Latin for “unique”.

##### Remark.

The holotype is the smaller of the two females in the vial containing the types and has the epigyne dissected.

##### Female

**(holotype, Chabal Mbabo, MRAC 162219).** Measurements: CL 2.68, CW 1.98, AL 3.50, AW 2.45, TL 5.85 (5.85–6.00), FL 0.21, SL 1.17, SW 1.15, AME–AME 0.08, AME–ALE 0.02, ALE–ALE 0.34, PME–PME 0.13, PME–PLE 0.06, PLE–PLE 0.55, PERW 0.81, MOQAW 0.33, MOQPW 0.46, MOQL 0.44.

Length of leg segments (sequence from femur to tarsus, and total): I 1.88 + 0.81 + 1.48 + 1.27 + 0.86 = 6.30; II 1.71 + 0.78 + 1.23 + 1.14 + 0.75 = 5.61; III 1.59 + 0.75 + 1.11 + 1.25 + 0.63 = 5.33; IV 2.25 + 0.93 + 1.95 + 2.10 + 0.85 = 8.08.

Carapace deep red-brown, clypeus brown, eye region darker; faint black striae radia- ting from fovea towards palps and leg coxae; surface finely granulate, densely covered in white plumose setae. AER procurved, ALE much larger than AME; AME separated by distance approximately ^3^∕_5_ their diameter, AME separated from ALE by ^1^∕_5_ AME diameter; clypeus height equal to 1½ AME diameter; PER procurved, PLE slightly larger than PME; PME separated by distance slightly less than ¾ their diameter, PME separated from PLE by distance slightly less than ⅓ PME diameter; CW:PERW = 2.44:1. Chelicerae brown with faint black mottling on anterior surface, yellow-orange along prolateral distal margin; three teeth on promargin, median tooth largest, distal tooth smallest, situated closest to median tooth; two teeth on retromargin, distal tooth slightly smaller than proximal tooth, closer to fang base than promarginal teeth; endites yellow-brown, cream prolaterally; labium orange-brown, cream distally; sternum orange with brown mottling. Legs finely granulate; femora I–IV dark brown, yellow dorsally at distal end; patellae I–IV yellow-brown, with dark brown mottling laterally; tibiae I and II yellow-brown, with sparse dark brown mottling laterally; tibiae III and IV dark brown, with paired pale brown stripes dorsally, distal ends bright yellow; metatarsi I and II yellow-brown, with sparse dark brown mottling laterally; metatarsi III and IV dark brown, yellow at proximal and distal ends; tarsi I and II yellow-brown, III and IV yellow. Leg spination: femora: I pl 1 do 3, II do 3, III pl 2 do 3 rl 1, IV pl 1 do 3 rl 1; patellae with do 1 long distal seta; tibiae: I plv 1 rlv 1, II plv 1 rlv 1, III pl 2 do 1 rl 2 plv 2 rlv 1 vt 2, IV pl 2 do 1 rl 2 plv 2 rlv 1 vt 2; metatarsi: I plv 2 rlv 2, II plv 2 rlv 2, III pl 3 rl 3 plv 2 rlv 2 vt 3, IV pl 3 rl 3 plv 2 rlv 2 vt 3. Palpal segments brown, tarsi orange-brown. Palpal spination: femora do 2, patellae pl 1 do 2, tibiae pl 1 do 1 plv 1, tarsi pl 1 plv 3 rlv 1. Abdomen mottled dark grey, with orange-brown dorsal scutum extending ¼ abdomen length; venter mottled pale grey, darker towards spinnerets, epigastric sclerite orange-brown and inframamillary sclerite yellow-brown. Epigyne with lateral copulatory openings situated within small round epigynal ridges (Fig. 93); copulatory ducts initially directed dorsally, looping transversely then anteriorly, entering ST II posterolaterally; ST II somewhat triangular, with sharply angled lateral margins, joined broadly to kidney-shaped posterior ST I (Fig. 94).

##### Male.

Unknown.

##### Distribution.

**Figure 95.**
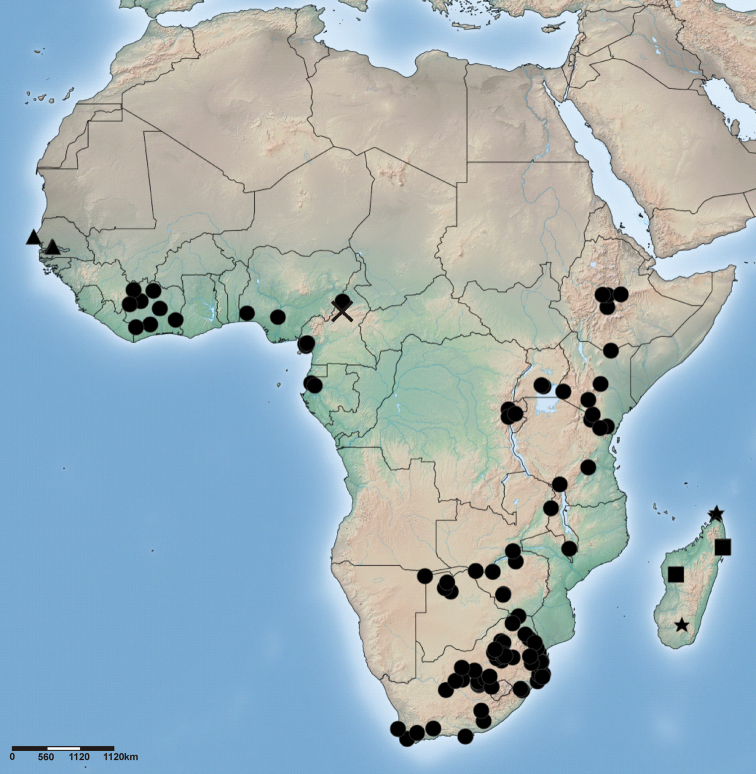
Distribution of *Cambalida fulvipes* (Simon, 1896) (circles), *Cambalida griswoldi* sp. n. (stars), *Cambalida lineata* sp. n. (squares), *Cambalida loricifera* (Simon, 1885) (triangles) and *Cambalida unica* sp. n. (cross) in the Afrotropical Region.

Only known from the type locality (Fig. 95).

##### Biology.

Unknown.

### Species *nomina dubia*

#### Cambalida insulana

Simon, 1909

Cambalida insulana Simon, 1909: 369.

##### Remarks.

The type material could not be traced in MNHN and is probably lost (Christine Rollard, pers. comm.), which was confirmed by Bosselaers and Jocqué (2000), who also could not successfully locate the type. The type locality given by Simon (1909) is “Ile Annobon”, also known as Pagalu Island, which is situated 160km south-west of Sao Tomé in the Atlantic Ocean. The only *Cambalida* material available from these islands is a series of immature and subadult specimens from Parc Nacional Ôhó, Sao Tomé, collected in 2001 and deposited in CAS. It is thus likely that *Cambalida insulana* populations may still be viable and not extinct. Additional adult material from the type locality is needed before this species can be validated, redescribed and neotypes designated.

#### Cambalida simoni nom. n.

Cambalida fulvipes Simon, 1909: 369 [preoccupied by senior homonym]

##### Remarks.

A replacement name is here proposed for *Cambalida fulvipes* Simon, 1909, a name occupied by its secondary senior homonym, *Cambalida fulvipes* (Simon, 1896), proposed earlier in this paper. The type material of this species could not be traced in MNHN and is probably lost, and the original description is inadequate for its successful identification. The occurrence of three different species in West Africa, and the lack of any comparable fresh material from Guinee-Bissau, from which this species was described, means that this species should remain *nomen dubium* until fresh material can be collected from the vicinity of the type locality (Bolama, 11°34'N, 15°29'W).

## Discussion

The current revision of the Afrotropical genus *Cambalida* increased the species compliment from three to ten species, with a further two species (including the type species of the genus) being considered *nomina dubia*. The biogeographical patterns of *Cambalida* are quite different to most of the other Afrotropical genera. *Apochinomma* Pavesi, 1881 and *Copa* Simon, 1885 (Haddad 2012) each have a single widespread Afrotropical species and *Echinax* Deeleman-Reinhold, 2001 (Haddad in press) has two widespread species, with the other species in each genus generally being restricted to a single biogeographical region (e.g. West Africa) or a single country. In contrast, *Cambalida* has one species widespread throughout the region (*Cambalida fulvipes*), two additional species widespread through tropical West and Central Africa (*Cambalida coriacea* and *Cambalida deminuta*), and one widespread in southern Africa (*Cambalida dippenaarae*). The remaining six species are all comparatively range restricted. While *Copa* has radiated considerably on Madagascar, with more than 30 new species to be described from the island (Haddad 2012), only two new Madagascan species of *Cambalida* have been described in this study. However, there is a large quantity of unidentified material from the island, and it is plausible that several new *Cambalida* have yet to be discovered. Until such time as the fauna has been more thoroughly studied, no discussion or hypotheses of the biogeographical relationships of the Madagascan and continental faunas should be made.

*Cambalida* appear to be exclusively ground-dwelling leaf litter spiders occurring mainly in savanna and forest habitats. In savannas they are generally uncommon but are similar in abundance to *Copa flavoplumosa* Simon, 1885 and *Merenius* spp. (Foord et al. 2008; Haddad et al. 2010; Muelelwa et al. 2010). In contrast, they contribute a more significant proportion of spider assemblages in the leaf litter of shrubs in the South African Grassland Biome, where they are the most abundant corinnids (Butler and Haddad 2011). They are only occasionally collected from agroecosystems in South Africa (Haddad and Dippenaar-Schoeman 2006), but several species are quite common in rice and fallow habitats in West Africa.

The current study has significantly increased the species compliment of *Cambalida* to ten, and it is likely that further new species will be sampled and need to be described in the future. This is supported by the reasonably small distribution ranges of several species (e.g. *Cambalida griswoldi*, *Cambalida lineata* and *Cambalida loricifera*) and others that are only known from the type locality (*Cambalida fagei* and *Cambalida unica*). It is probable that many historically poorly sampled biodiversity hotspots may yield considerable additions to the fauna, particularly in East and West Africa. The possible occurrence of *Cambalida* on the Indian Ocean islands (other than Madagascar) also requires further investigation.

## Supplementary Material

XML Treatment for Cambalida

XML Treatment for Cambalida compressa

XML Treatment for Cambalida coriacea

XML Treatment for Cambalida deminuta

XML Treatment for Cambalida dippenaarae

XML Treatment for Cambalida fagei

XML Treatment for Cambalida fulvipes

XML Treatment for Cambalida griswoldi

XML Treatment for Cambalida lineata

XML Treatment for Cambalida loricifera

XML Treatment for Cambalida unica

XML Treatment for Cambalida insulana

XML Treatment for Cambalida simoni
